# Rash morphology as a predictor of COVID‐19 severity: A systematic review of the cutaneous manifestations of COVID‐19

**DOI:** 10.1002/ski2.120

**Published:** 2022-05-12

**Authors:** Zack Holmes, Ashling Courtney, Marc Lincoln, Richard Weller

**Affiliations:** ^1^ Department of Medicine St. Vincent’s Hospital Melbourne Victoria Australia; ^2^ Department of Dermatology Perth Children’s Hospital Perth Western Australia Australia; ^3^ Department of Medicine St. James’ Hospital Dublin Ireland; ^4^ Department of Dermatology University of Edinburgh Edinburgh UK

## Abstract

Approximately 6% of those with COVID‐19 will experience cutaneous manifestations. Examining data from this cohort could provide useful information to help with the management of COVID‐19. To that end, we conducted a systematic review primarily to assess rash morphologies associated with COVID‐19 and their relationship with disease severity. Secondary outcomes include demographics, distribution, dermatological symptoms, timeline, diagnostic method and medication history. The literature was searched for all patients with skin manifestations thought to be related to suspected or confirmed COVID‐19. Patients with a history of dermatological, rheumatological or occupational skin disorders were excluded. Of the 2056 patients selected, the most common morphologies were chilblain‐like lesions (54.2%), maculopapular (13.6%) and urticaria (8.3%). Chilblain‐like lesions were more frequent in the younger population (mean age 21.5, standard deviation ± 10.8) and were strongly linked with milder disease, not requiring an admission (odds ratio [OR] 35.36 [95% confidence interval {CI} 23.58, 53.03]). Conversely, acro‐ischaemia and livedo reticularis were associated with worse outcomes, including a need for ICU (OR 34.01 [95% CI 16.62, 69.57] and OR 5.57 [95% CI 3.02, 10.30], respectively) and mortality (OR 25.66 [95% CI 10.83, 60.79] and OR 10.71 [95% CI 4.76, 24.13], respectively). Acral lesions were the most common site (83.5%). 35.1% experienced pruritus, 16.4% had pain and 4.7% reported a burning sensation. 34.1% had asymptomatic lesions. Rash was the only symptom in 20.9% and occurred before or alongside systemic symptoms in 12.4%. 28.3% had a positive polymerase chain reaction nasopharyngeal swab and 5.4% had positive antibodies, while 21.9% tested negative and 45.1% were not tested. In conclusion, COVID‐19 causes a variety of rashes, which may cause symptoms and add to morbidity. Rash type could be helpful in determining COVID‐19 prognosis.

## Graphical abstract

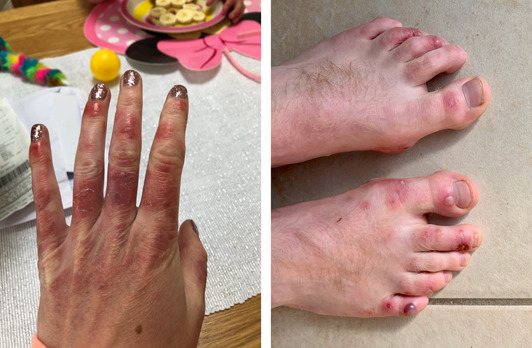

1

**What is already known about this topic?**

- COVID‐19 is a complex disease that can affect multiple systems.
- Clinical features are important in the management of COVID‐19.
- There are increasing reports of cutaneous manifestations of COVID‐19 in the literature.
- COVID‐19 appears to cause a wide variety of rash morphologies.
- Little is known about the relationship between rash type and COVID‐19 severity.

**What does this study add?**

- An updated summary of the cutaneous manifestations of COVID‐19.
- A detailed discussion of characteristics associated with morphologies including dermatological symptoms and timeline.
- Evidence that rash type may be of prognostic value in the management of COVID‐19.
- Rash may be the only symptom of COVID‐19, as was seen in 20.9% of patients in our review.

## 2 INTRODUCTION

COVID‐19, caused by the severe acute respiratory syndrome coronavirus 2 (SARS‐Cov‐2), has spread rapidly through human to human transmission worldwide since its first identification in Wuhan, China, in December of 2019. The World Health Organisation (WHO) declared it a pandemic in March 2020 and as of November 2021, an estimated 250 million people have been infected, resulting in over 5 million deaths.^1^ While primarily affecting the respiratory tract, COVID‐19 is known to also affect multiple other systems, including the skin. Initial reports from China put the incidence of skin manifestations at 0.2%, while data from the ZOE COVID Symptom Study app estimated the incidence among over 4 million self‐reporting contributors to be approximately 9%.^2^, ^3^ A study from Italy where patients were screened by Dermatologists found skin changes in 20% of inpatients infected with COVID‐19, while a large systematic review and meta‐analysis estimated the overall prevalence of cutaneous manifestations in COVID‐19 patients is 5.69%.^4^, ^5^ As a result of increased awareness of the cutaneous manifestations of COVID‐19, the amount of literature published on the topic has grown exponentially since the pandemic began. Closely analysing this data may allow us to learn more about the disease process and investigate whether cutaneous findings could provide useful information to help with diagnosis and prognosis. This is especially important with regard to allocation of medical resources and informing the pre‐test probability, given the virus' highly infectious nature and logistical factors affecting testing availability and time from test to result. To that end, this article summarizes the publications related to the cutaneous manifestations of COVID‐19 infection with the primary outcomes of rash morphology and COVID‐19 severity. Secondary outcomes include demographics, rash distribution, symptoms, timing, diagnostic method and medication history.

## 3 METHODS

### 3.1 Study selection

A systematic review of peer‐reviewed and pre‐print published literature was conducted according to the Preferred Reporting Items for Systematic Reviews and Meta‐Analyses (PRISMA) guidelines. The Medline database (PubMed; https://pubmed.ncbi.nlm.nih.gov/), medRxiv (https://www.medrxiv.org/) and bioRxiv (https://www.biorxiv.org/) were searched using the search terms ‘((coronavirus) OR (SARS‐CoV‐2) OR (COVID) OR (COVID‐19)) AND ((dermatology) OR (dermatological) OR (skin) OR (cutaneous) OR (rash) OR (dermis) OR (epidermis)). The search was performed on 5 February 2021 and limited to papers published up until 31 December 2020 in order to select for an unvaccinated cohort with the same or similar variants. Additional articles were obtained from reviewing bibliographies. Duplicates were removed and potential studies were selected after screening of title and abstract by two investigators independently.

### 3.2 Selection criteria

The inclusion criteria were as follows: (i) Population: patients with skin changes in the context of confirmed or suspected COVID‐19 infection. Patients with cutaneous manifestations thought to be related to COVID‐19 in the absence of other more typical symptoms such as cough, rhinorrhoea, fever, and so on, were also included. (ii) Design: case reports, case series, case control studies and cohort studies.

The exclusion criteria encompassed papers not in English, review articles and those with a lack of clinical data. Patients were excluded if they had a history of dermatological or rheumatological disease, including chilblains, iatrogenic lesions such as pressure sores and occupational skin disorders such as personal protective equipment (PPE) related rashes. Reactivated skin conditions, such as herpes simplex virus ulcers, were also excluded. Cases where a second pathological agent was found and those where a drug reaction was confirmed were also excluded. Efforts were made to ensure studies were not double counted and articles such as case series were screened for inclusion of cases already published or included in this review. Within each study, where some patients met the criteria for inclusion and others for exclusion, those were included in the data collection where possible. When these patients could not be reliably separated, due to issues such as the way the data were presented, the entire study was excluded.

### 3.3 Data extraction

Details such as digital object identifier, journal, study design, country, skin type, gender, mean age, rash morphology, distribution of lesions, dermatological symptoms, rash timing, COVID‐19 diagnostic method, COVID‐19 disease severity and recent medication changes were extracted from the selected studies as per the primary and secondary outcomes.

**FIGURE 1:**
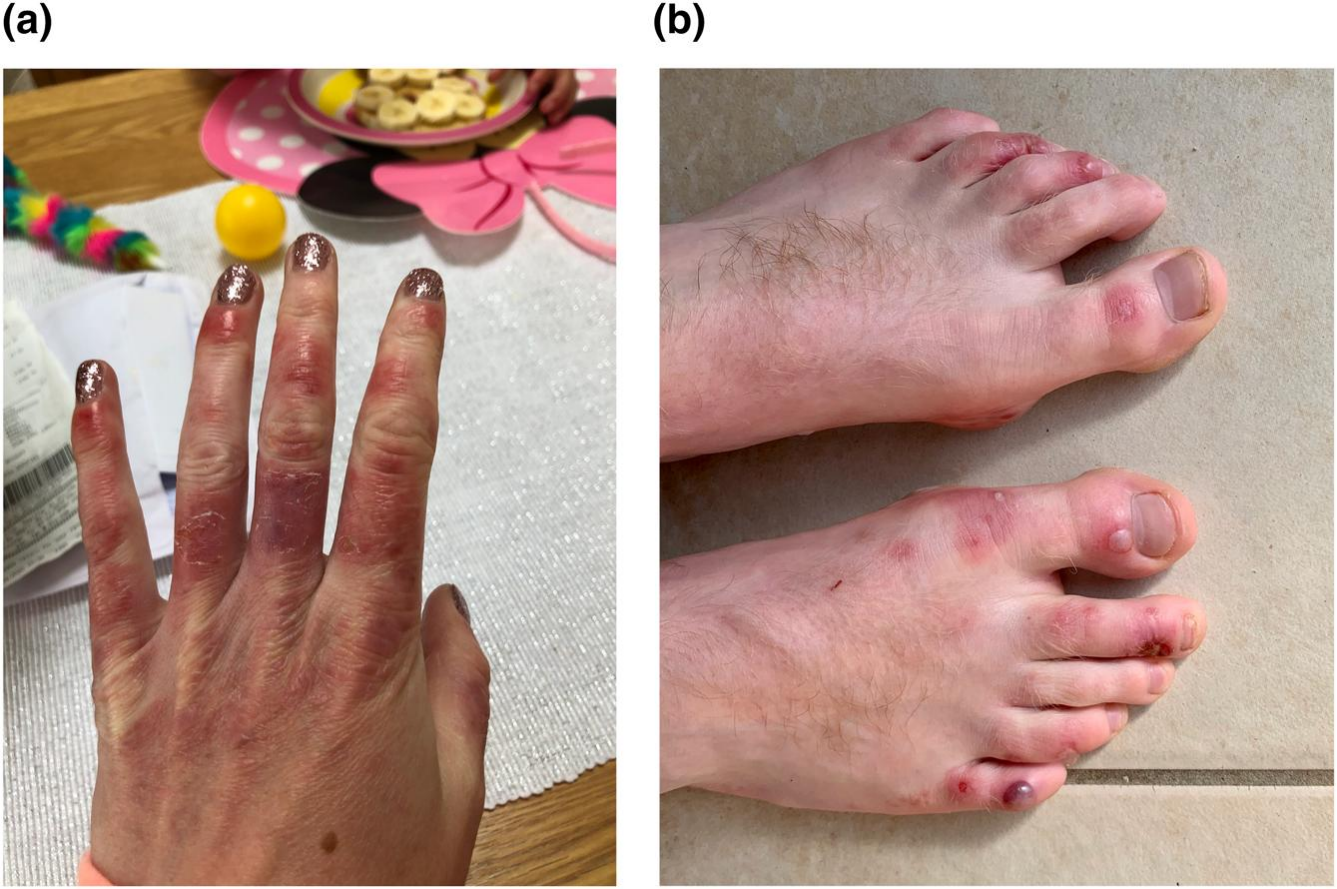
Chilblain‐like lesions on the hand and foot of patients with COVID‐19. Reproduced with permission from Zoe Global Ltd

**FIGURE 2:**
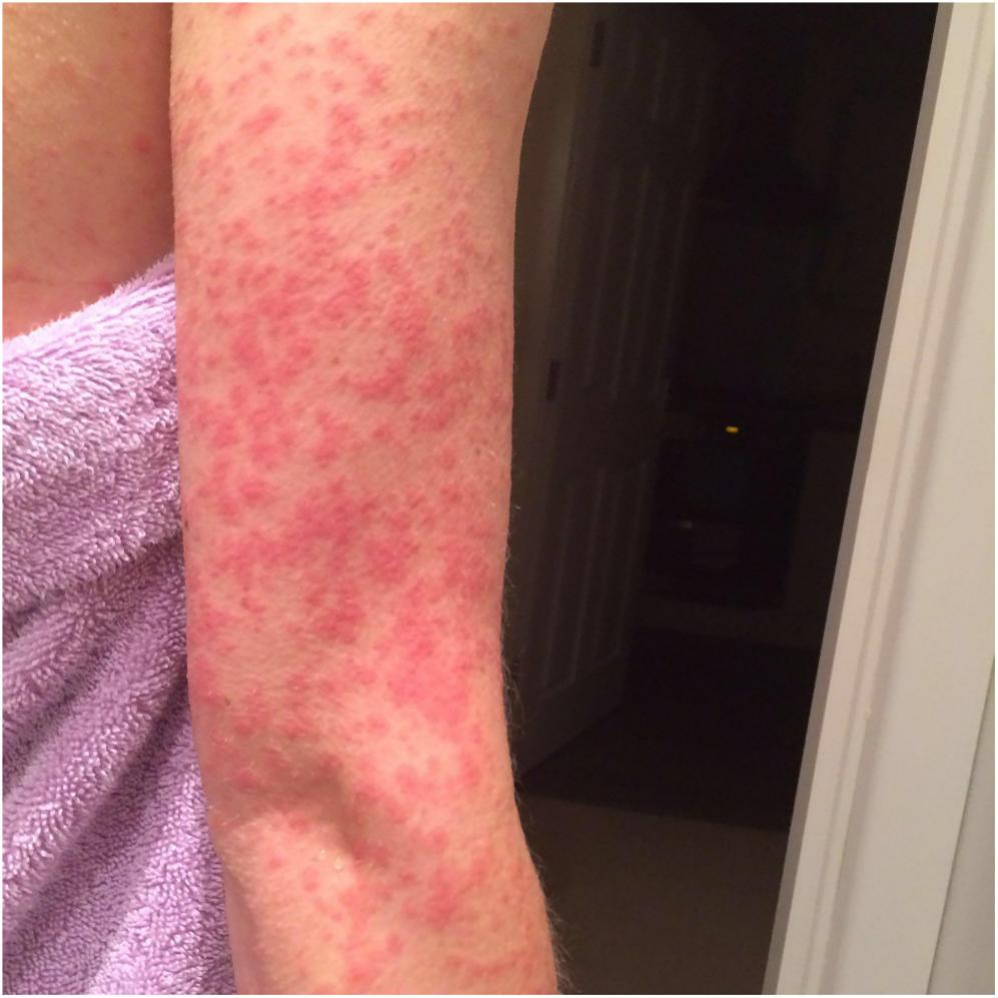
Maculopapular rash on the arm of a patient with COVID‐19. Reproduced with permission from Zoe Global Ltd

**FIGURE 3:**
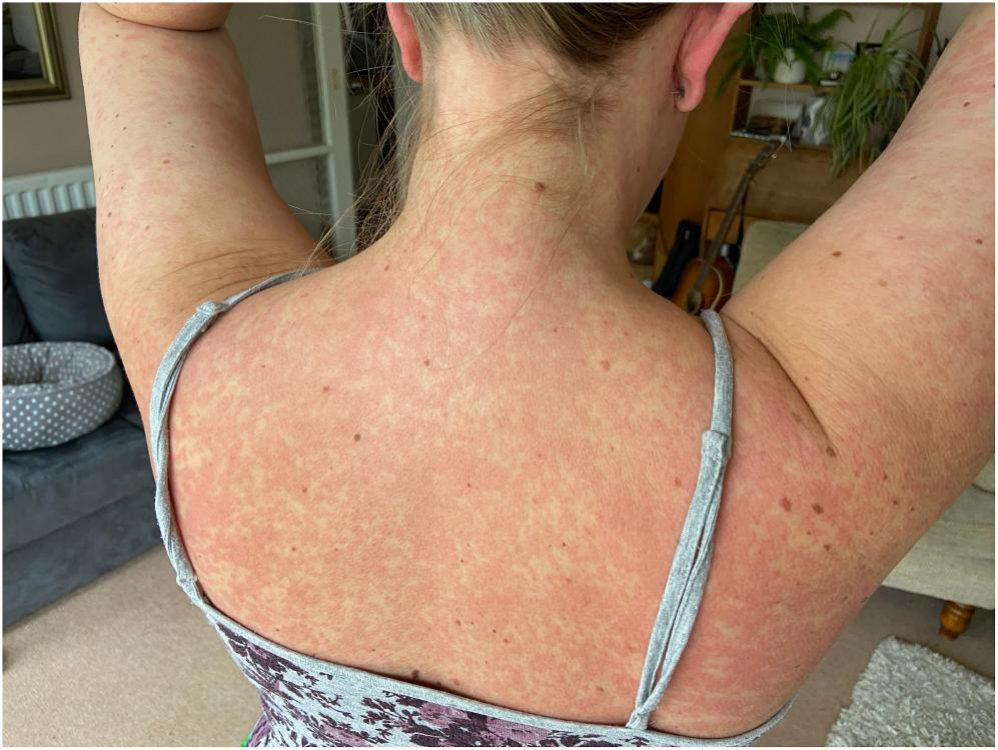
Generalized erythematous rash of a patient with COVID‐19. Reproduced with permission from Zoe Global Ltd

**FIGURE 4:**
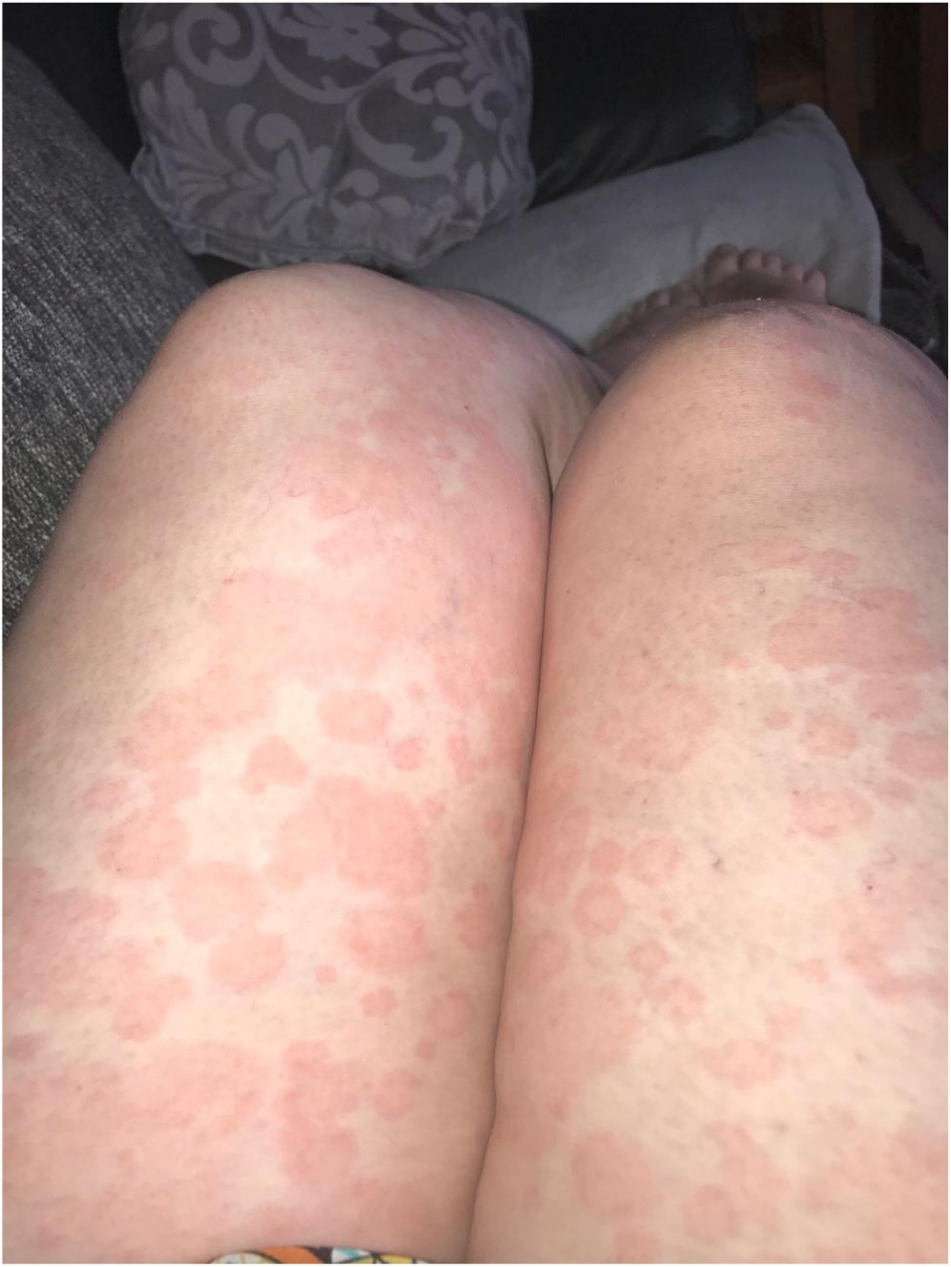
Urticarial rash on the legs of a patient with COVID‐19. Reproduced with permission from Zoe Global Ltd

**FIGURE 5:**
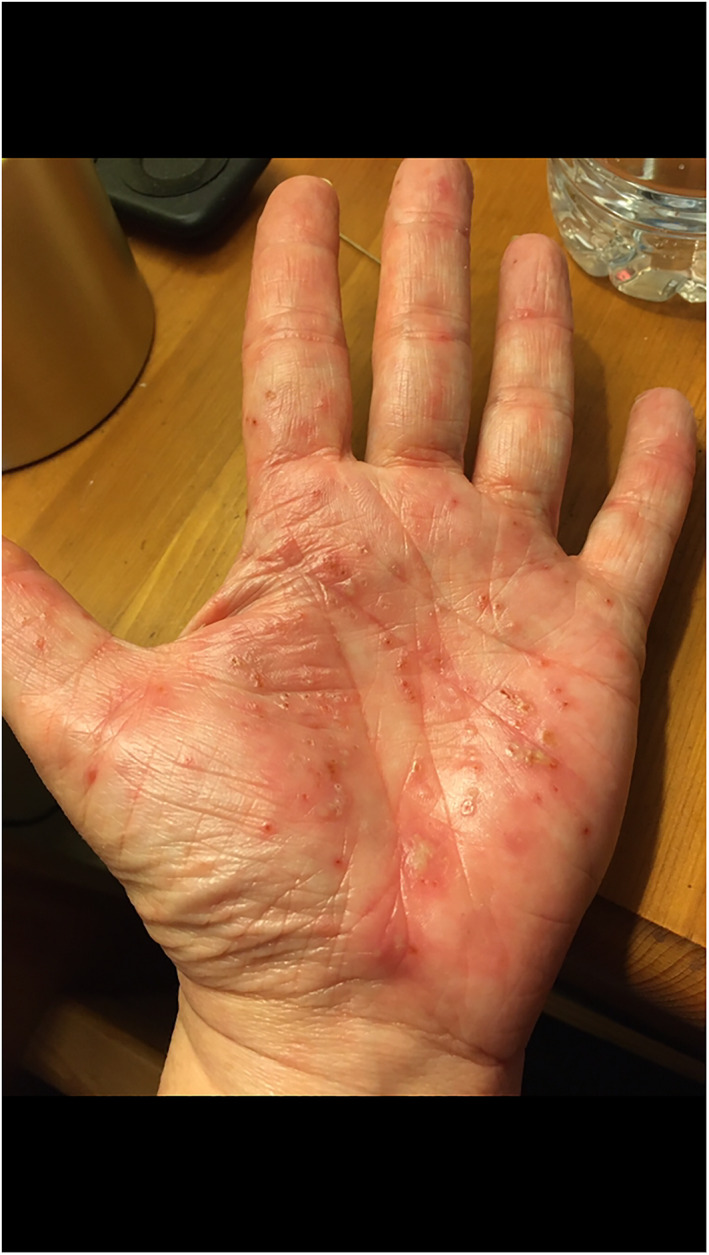
Vesiculobullous rash on the palm of a patient with COVID‐19. Reproduced with permission from Zoe Global Ltd

**FIGURE 6:**
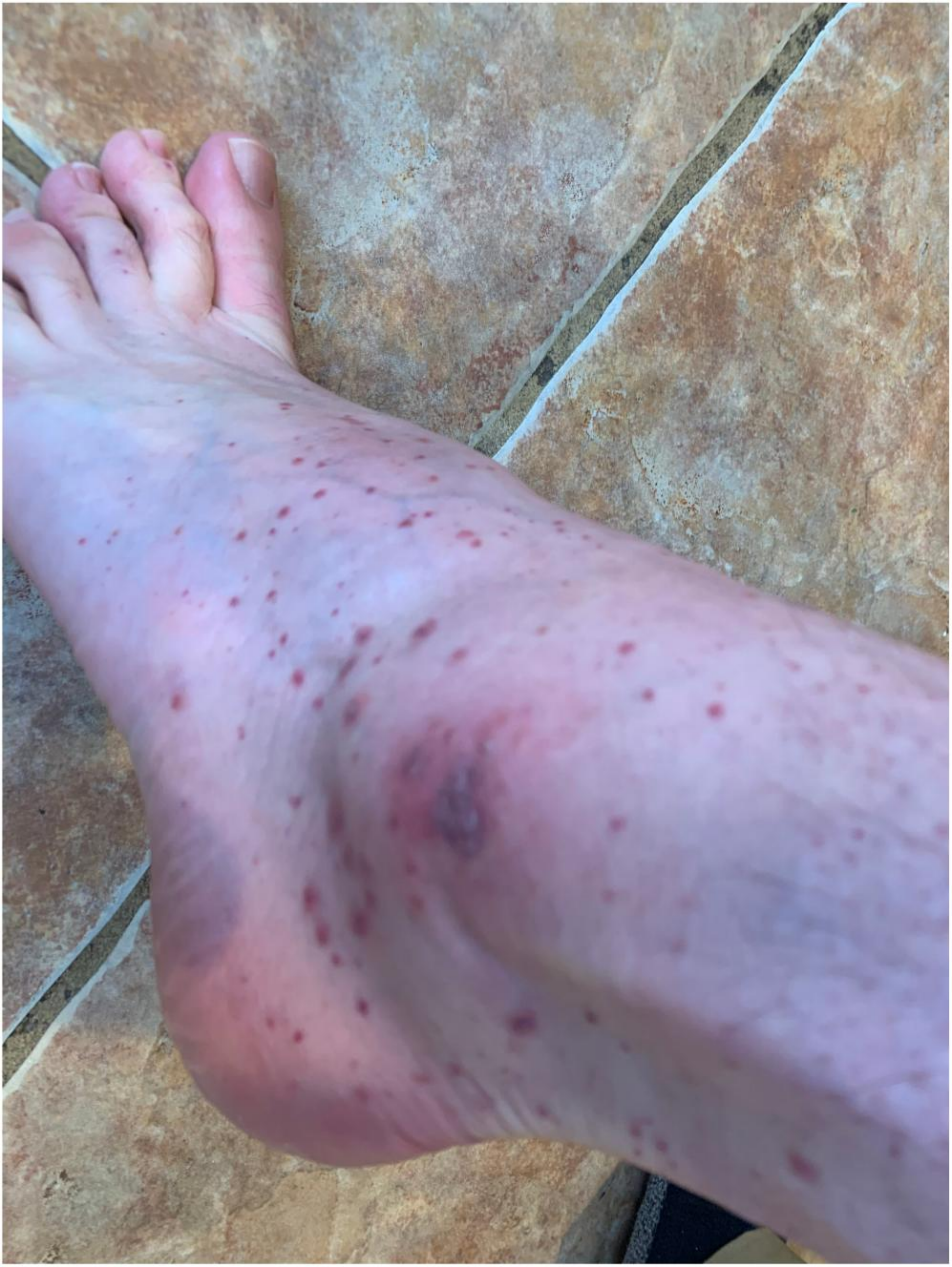
Purpuric rash on the ankle of a patient with COVID‐19. Reproduced with permission from Zoe Global Ltd

**FIGURE 7:**
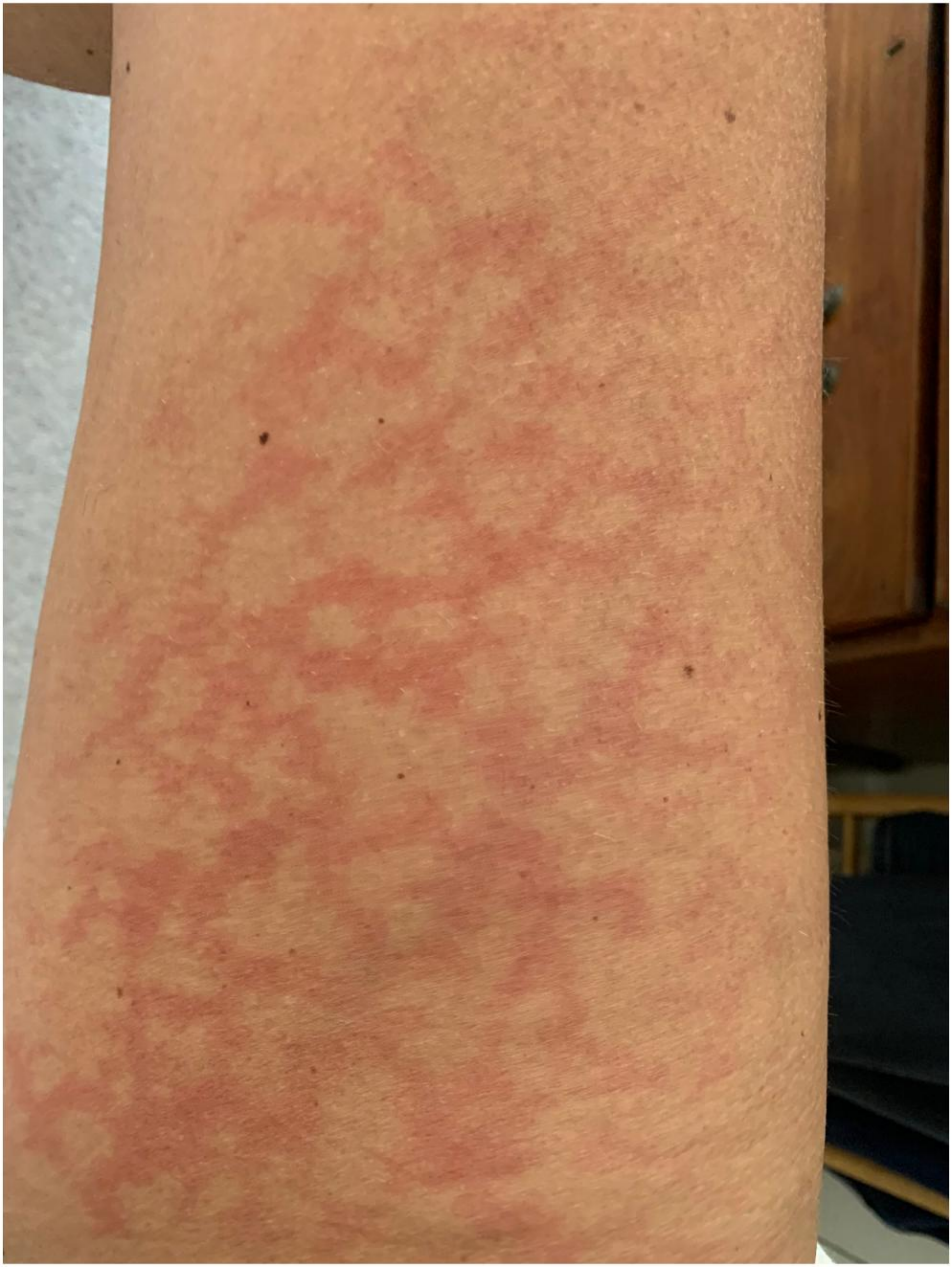
Livedo reticularis on the thigh of a patient with COVID‐19. Reproduced with permission from Zoe Global Ltd

Rash morphologies were recorded as Chilblain‐like (Figure 1a,b), Maculopapular (Figure 2), Erythematous (Figure 3), Urticarial (Figure 4), Vesiculobullous (Figure 5), Purpuric (Figure 6), Livedo reticularis (Figure 7), Acro‐ischaemic, Erythema multiforme‐like, Mouth ulcers, Hair and Nail changes and Other. Currently, there are no standardized criteria regarding the cutaneous manifestations of COVID‐19. These rash types were chosen as the categories for this study based on the general consensus among articles published to date, with the majority of morphologies fitting into one of these subtypes.

Distribution of lesions was recorded as ‘Acral’, ‘Limbs’, ‘Head and Neck’, ‘Mucosal’, ‘Trunk’, ‘Generalized’ and ‘Hair and Nails’.

Dermatological symptoms were recorded as ‘Pain’, ‘Pruritus’, ‘Burning’ or ‘Asymptomatic’.

The timeline of skin changes relative to systemic symptoms was recorded as: ‘Only symptom’, ‘First Symptom’, ‘First Cluster’, ‘Rash Onset’ and ‘Rash Duration’.

COVID‐19 diagnostic method was recorded as Polymerase Chain Reaction (PCR) positive, Antibody positive, Negative test, Nil test (a), Nil test (b), or method not stated.

COVID‐19 severity was classified as ‘Outpatient’, ‘Inpatient’, ‘Intensive Care Unit’ (ICU) or ‘Died’ (RIP).

Medication history was recorded as either ‘New Medication’ or ‘No Medication Changes’.

## 4 DEFINITION OF OUTCOME PARAMETERS

### 4.1 Outcome parameters related to clinical morphology

Chilblain‐like: erythematous‐oedematous, erythematous‐violaceous, blistering lesions over fingers and toes; Maculopapular: maculopapular or morbilliform rashes; Erythematous: erythematous macules or patches not fitting other morphologies; Urticarial: urticaria or wheals; Vesiculobullous: blisters, vesicles, bullae, herpes‐like, varicella‐like or papulovesicular lesions; Purpuric: purpura, ecchymoses, bruises, petechiae; Livedo reticularis: livedo reticularis, livedo racemosa, reticular purpura or mottling of skin; Acro‐ischaemia: blue/black discolouration and gangrene of fingers and toes; Erythema multiforme‐like: erythema multiforme, target‐like lesions or targetoid lesions; Oral ulcers: ulcers present on oral mucosal surfaces; Hair and Nail changes: alopecia, anagen/telogen effluvium or any nail change not attributable to any other cause; Other: skin changes not compatible with other categories.

### 4.2 Outcome parameters related to the distribution of lesions

Acral: lesions limited to the hands, fingers, feet and toes without involvement of other sites; Limbs: lesions limited to arms, forearms, thighs, legs or glutaeal region without the involvement of other sites; Head and neck: lesions limited to the face including forehead, cheeks, pre‐ and postauricular areas, chin and neck without the involvement of eyes, mouth, hair or other sites; Mucosal: lesions limited to oral mucosa without involvement of ocular mucosa or other sites; Trunk: lesions limited to chest, abdomen and back without the involvement of other sites; Generalized: widespread rash; Hair and nails: lesions limited to hair and nails without involvement of other sites. Patients may be included in more than one category.

### 4.3 Outcome parameters related to dermatological symptoms

Pain: painful sensation, not including burning, related to the skin changes; Burning: burning sensation related to the skin changes; Pruritus: itch; Asymptomatic; specific mention of asymptomatic skin lesions.

### 4.4 Outcome parameters related to rash timeline

Only symptom: rash was the only symptom reported; First symptom: rash was the first symptom and other systemic symptoms subsequently developed; First cluster: rash occurred at the same time as other systemic symptoms; Rash onset: timing of rash relative to other systemic symptoms; Rash duration: length of time from rash onset to complete resolution.

### 4.5 Outcome parameters related to COVID‐19 diagnostic method

PCR positive: positive result from nasopharyngeal reverse transcriptase PCR swab; Antibody positive: positive result from COVID‐19 serum antibodies including immunoglobulin A, E, G and M (IgA, IgE, IgG and IgM); Negative test: negative result from either PCR swab or serum antibodies and no subsequent positive test. Patients who had a negative PCR swab and subsequently had positive serum antibodies were included as Antibody positive and not as Negative test. Patients with a positive PCR and antibodies were included in both categories. Nil test (a); not tested, but had COVID‐19 risk factors such as suggestive symptoms or a positive close contact; Nil test (b): not tested and had no COVID‐19 risk factors, but had a rash thought to be related to COVID‐19; Method not stated: patients stated to be diagnosed with COVID‐19, but the method is not specified.

### 4.6 Outcome parameters related to COVID‐19 disease severity

Patients were counted in a single category, which was the maximum level of severity associated with that case.

### 4.7 Outcome parameters related to medication history

New medication: commenced treatment for COVID‐19 or its complications prior to rash onset; No medication changes: no recent medication changes.

### 4.8 Statistical analysis

Data were recorded on a spreadsheet using the software package Numbers. Graphs were made using a combination of Numbers and Microsoft Excel.

A chi‐squared test was performed for each rash morphology to examine its relationship with severity outcomes. The null hypothesis for this test was that there is no relationship between rash type and outcome and therefore, expected numbers should be evenly distributed across all four severity outcomes. A two‐tailed test was performed, with 3 degrees of freedom and a chosen alpha value of 0.05.

Odds ratios (ORs), with accompanying confidence intervals (CIs), were calculated for each rash type and severity outcome using the 2 × 2 table method, with rash type and all other rashes plotted against severity outcome and all other severity outcomes.

With regard to proportions, only complete data were used to account for study heterogeneity. Binomial CIs for population proportions were calculated using the Clopper–Pearson method. A direct acyclic graph (DAG) was made to illustrate causal inference and the effect of confounders on the relationship between COVID‐19 as the exposure and the outcomes of rash and severity. Subgroup analysis was performed on the confounders age and medications in an attempt to minimize heterogeneity.

A sensitivity analysis was performed using only papers with non‐selective reporting of both presence and type of cutaneous signs in order to validate whether our study sample was an accurate reflection of the true population.

## 5 RESULTS

### 5.1 Study selection and study population

**FIGURE 8:**
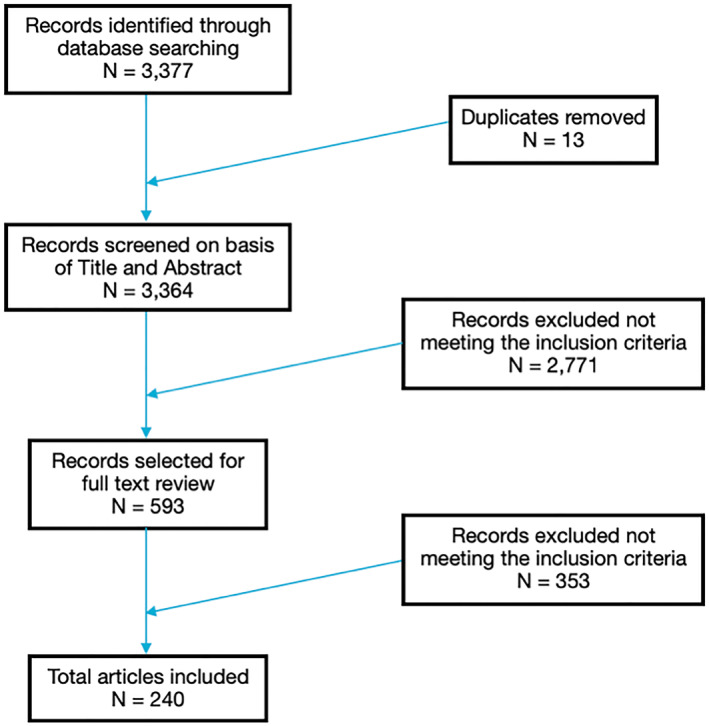
PRISMA flow diagram showing literature search and article selection

A total of 3377 papers were identified from the search, of which 2572 were from PubMed and 805 were from medRxiv and bioRxiv. Thirteen of those papers were duplicates and therefore removed. The remaining papers were then assessed further by title and abstract screening and following this, 593 articles were selected for full‐text review. Finally, 240 papers met the pre‐specified criteria and were included, comprising of 154 case reports and 86 observational studies. This is illustrated in the PRISMA flow diagram (Figure 8). Most articles were from Spain (47), America (42), Italy (36) and France (20).

### 5.2 Basic information

In total, 2056 patients were included, with 883 males (50.8%) and 855 females (49.2%). The mean age of 1285 patients was 35.3 (standard deviation ± 19.4, range: 0–100 years), which is younger compared to early reviews published in China, which calculated a mean age of 49.54.6 Skin type reporting was infrequent and documented in only 353 patients. Of these patients, 293 (83.0%) were labelled ‘White’, while 22 (6.2%) were ‘Asian’, 20 (5.7%) were ‘Hispanic’, 6 (1.7%) were ‘Black’ and 1 (0.3%) was ‘Indian’. For those patients referred to as ‘Asian’, no further information was available. Two patients were stated to be Fitzpatrick type 3, while seven were type 4 and two were type 5. It was not possible to ascertain patient skin type from clinical photography of cutaneous lesions.

### 5.3 Clinical morphology of cutaneous manifestations of COVID‐19 patients

The most frequently described cutaneous manifestation was chilblain‐like lesions, which occurred in 1115 (54.2%) patients. This predominance is in stark contrast to reviews published early in the pandemic from China, which reported erythema (44.2% and 38.4%) was the most common finding, while chilblain‐like lesions were seen much more infrequently (19.7% and 10.1%).^6^, ^7^ Maculopapular was the second most commonly seen type (13.6%), followed by urticaria (8.3%). A complete breakdown of morphology frequency is summarized in Table 1. Some patients displayed more than one type of lesion and were therefore counted in multiple categories.

**TABLE 1:** Primary outcomes of rash morphology and COVID‐19 disease severity

Outcome	Total	Proportion (%)	95% CI	Chi‐squared
Morphology (*n* = 2056)				
(a) Chilblain‐like	1115	54.2	52.1, 56.4	*p* < 0.001
(b) Maculopapular	280	13.6	12.2, 15.2	*p* < 0.001
(c) Urticaria	170	8.3	7.1, 9.5	*p* < 0.001
(d) Vesiculobullous	127	6.2	5.2, 7.3	*p* < 0.001
(e) Erythema	81	3.9	3.1, 4.9	*p* < 0.001
(f) Purpura	70	3.4	2.7, 4.3	*p* < 0.001
(g) EM‐like	68	3.3	2.6, 4.2	*p* < 0.001
(h) Livedo reticularis	49	2.4	1.8, 3.1	*p* < 0.001
(i) Acro‐ischaemia	37	1.8	1.3, 2.5	*p* < 0.001
(j) Hair and nail	22	1.1	0.7, 1.6	*p* < 0.001
(k) Oral ulcers	15	0.7	0.4, 1.2	*p* = 0.004
(l) Other	82	4.0	3.2, 4.9	
Severity (*n* = 1763)				
(a) OP	1272	72.1	70.0, 74.2	
(b) IP	365	20.7	18.8, 22.7	
(c) ICU	94	5.3	4.3, 6.5	
(d) RIP	32	1.8	1.2, 2.6	

### 5.4 Skin manifestations and COVID‐19 severity

Data on COVID‐19 severity was available for 1763 patients. 1272 (72.1%) had outpatient level severity, 365 (20.7%) required inpatient management, 94 (5.3%) were admitted to ICU and 32 (1.8%) died as a result of COVID‐19 or its complications. These results are summarized in Table 1. Few systematic reviews previously published have examined the relationship between cutaneous manifestations and COVID‐19 severity in detail. One review found the mortality rate to be 2.56% in those with COVID‐19 and a rash, but it did not provide any information on the other degrees of severity discussed in this study.^6^

**FIGURE 9:**
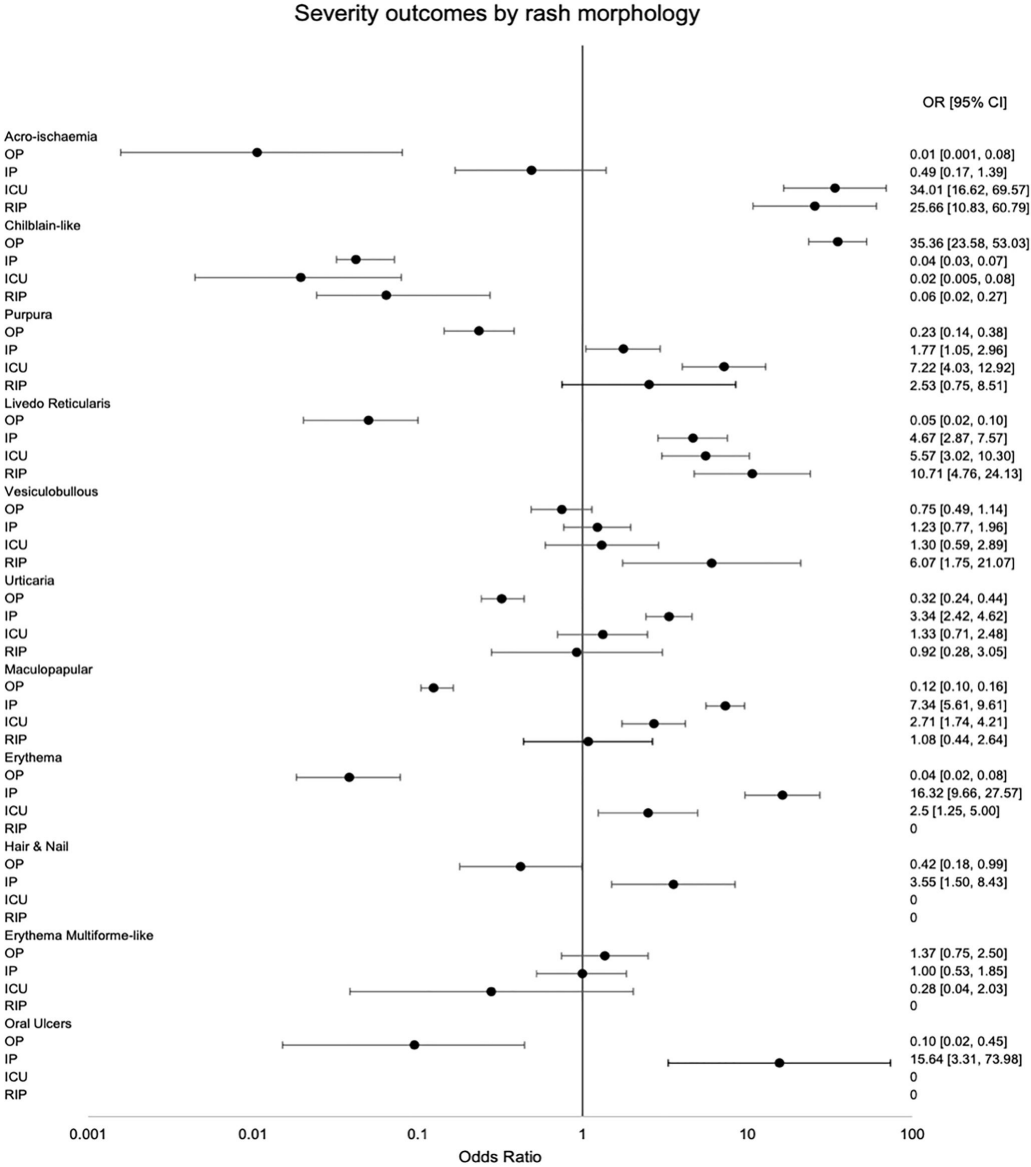
Forest plot demonstrating odds ratios and corresponding 95% confidence intervals for severity outcomes associated with each rash morphology

*Χ* ^2^ tests were statistically significant for each rash morphology group, indicating that there was not an even distribution of patients among the four severity outcomes in all groups. The levels of confidence for each test are included in Table 1. ORs and their corresponding 95% CIs were calculated for severity outcomes in each rash morphology and are shown on the Forest plot (Figure 9).

## 6 SECONDARY OUTCOMES

### 6.1 Distribution of skin lesions

This included 1150 patients, of whom 960 (83.5%) had acral involvement. Of those 960 patients, 700 had foot involvement, 71 had hand involvement and 89 had both hand and foot involvement. Trunk was the second most common site and was affected in 157 (13.7%) patients. Limbs were involved in 136 (11.8%), with 60 of those having both limbs affected, 40 having just upper limb involvement and the remaining 36 had lesions confined to their upper limbs. Forty‐nine (4.3%) had a generalized rash, while 44 (3.8%) had head and neck lesions, 30 (2.6%) had oral manifestations and 22 (1.9%) had hair and nail changes.

**TABLE 2:** Secondary outcomes including rash distribution, dermatological symptoms, rash timing with respect to systemic symptoms, diagnostic method and medication history

Outcome	Total	Proportion (%)	95% CI
Distribution (*n* = 1150)			
(a) Acral	960	83.5	81.2, 85.6
(b) Trunk	157	13.7	11.7, 15.8
(c) Limbs	136	11.8	10.0, 13.8
(d) Generalized	49	4.3	3.2, 5.6
(e) Head and neck	44	3.8	2.8, 5.1
(f) Oral mucosa	30	2.6	1.8, 3.7
(g) Hair and nail	22	1.9	1.2, 2.9
Dermatological symptoms (*n* = 700)			
(a) Pruritus	246	35.1	31.6, 38.1
(b) Pain	115	16.4	13.8, 19.4
(c) Burning	33	4.7	3.3, 6.6
(d) Asymptomatic	239	34.1	30.6, 37.8
Rash timing (*n* = 508)			
(a) 1st symptom	29	5.7	3.9, 8.1
(b) 1st cluster	34	6.7	4.7, 9.2
(c) Only symptom	106	20.9	17.4, 24.7
Diagnostic method (*n* = 1083)			
(a) PCR +ve	306	28.3	25.6, 31.0
(b) Ab +ve	59	5.4	4.2, 7.0
(c) −ve test	237	21.9	19.5, 24.5
(d) Nil test	488	45.1	42.1, 48.1
New medications (*n* = 578)			
(a) Yes	154	26.6	23.1, 30.5
(b) No	424	73.4	69.6, 76.9

### 6.2 Dermatological symptoms associated with skin lesions

Of 700 patients with complete data, 246 (35.1%) reported pruritus, 115 (16.4%) had pain, 33 (4.7%) experienced burning and 239 (34.1%) had asymptomatic lesions. Some patients had multiple symptoms and were included in more than one category.

### 6.3 Timeline

Among 508 patients with complete data, rash was the first symptom in 29 (5.7%) patients, part of the first cluster of symptoms in 34 (6.7%) patients and the only symptom in 106 (20.9%) patients. The mean onset of skin changes was 13.85 days and the mean duration was 13.16 days.

### 6.4 Diagnostic method of COVID‐19

There was complete data available for 1083 patients. Three hundred and six (28.3%) had a positive swab, while 59 (5.4%) had positive antibodies. 488 (45.1%) did not receive testing, despite 159 of them having COVID‐19 risk factors. Two hundred and thirty‐seven (21.9%) patients tested negative. Overall data for each secondary outcome is shown on Table 2.

## 7 MEDICATIONS

A total of 454 patients had recently received treatment for COVID‐19 before the development of skin signs. One hundred and nine received antibiotics, the most common of these being Azithromycin (44), Ceftriaxone (23), Tazocin (10) and Linezolid (6). Fifty‐six patients were treated with hydroxychloroquine and 43 received antivirals, including Lopinavir/Ritonavir (24), Oseltamivir (6), Favirapir (4), Darunavir, Ritonavir and Remdesevir (2 each). Twenty‐three patients received paracetamol, 19 patients were treated with corticosteroids, 21 were given heparin and 18 took supplements such as vitamin C, zinc, B12, thiamine and folate. Four hundred and twenty‐four patients had no recent medications commenced. Of the 578 patients with complete data, 154 (26.6%) took a new drug before rash onset, while the other 424 (73.4%) patients had no recent medication changes.

### 7.1 Secondary outcomes by rash type

Total data for each secondary outcome are arranged by rash type in Table 3.

**TABLE 3:** Table summarizing secondary outcomes for each rash morphology

Outcome	CB‐like	MP	U	VB	E	P	EM‐like	LR	AI	H&N	MU
Total	1115	280	170	127	81	70	68	49	37	22	15
Mean age (years)	21.46	51.74	45.85	49.52	34.08	43.73	34.08	55.60	58.43	52.96	37.79
Distribution											
(a) Acral	875	4	2	7	11	5	48	11	37	0	0
(b) Trunk	0	52	21	39	24	16	0	4	0	0	0
(c) Limbs	0	35	28	11	55	21	9	11	0	0	0
(d) Generalized	0	20	32	2	4	16	5	0	0	0	0
(e) Head and neck	3	11	6	3	7	3	0	1	0	0	0
(f) Oral mucosa	0	0	0	4	7	3	9	0	0	0	15
(g) Hair and nail	0	0	0	0	0	0	0	0	0	22	0
Dermatological symptoms											
(a) Pruritus	285	126	100	42	10	23	9	5	0	1	1
(b) Pain	346	7	3	8	3	13	6	2	5	0	7
(c) Burning	244	10	3	5	0	0	0	2	0	1	0
(d) Asymptomatic	138	80	7	20	8	23	2	17	3	20	1
Rash timing											
(a) 1st symptom	50	13	12	8	4	1	0	2	0	0	0
(b) 1st cluster	46	116	48	23	9	4	4	19	0	0	4
(c) Only symptom	464	1	6	1	1	2	1	0	1	0	1
(d) Onset (days)	15.9	10.6	7	5.6	9	11.7	8.5	14.1	23	50	3.4
(e) Duration (days)	16	8.9	6.7	9.4	7.1	8.5	13.4	8	32	‐	10
Diagnostic method											
(a) PCR +ve	30	66	57	42	69	38	10	15	24	17	11
(b) Ab +ve	44	8	2	2	7	4	2	1	1	5	1
(c) −ve test	250	1	2	1	1	2	4	2	1	0	0
(d) Nil test	505	55	27	18	2	1	0	4	0	0	1
New medications											
(a) Yes	42	186	70	26	17	16	10	29	17	12	3
(b) No	266	12	19	34	7	27	48	2	1	7	4

Abbreviations: AI, acro‐ischaemia; CB, chilblain; E, erythema; EM, erythema multiforme; H&N, head and neck; LR, livedo reticularis; MP, maculopapular; MU, mouth ulcers; P, purpura; U, urticaria; VB, vesiculobullous.

**FIGURE 10:**
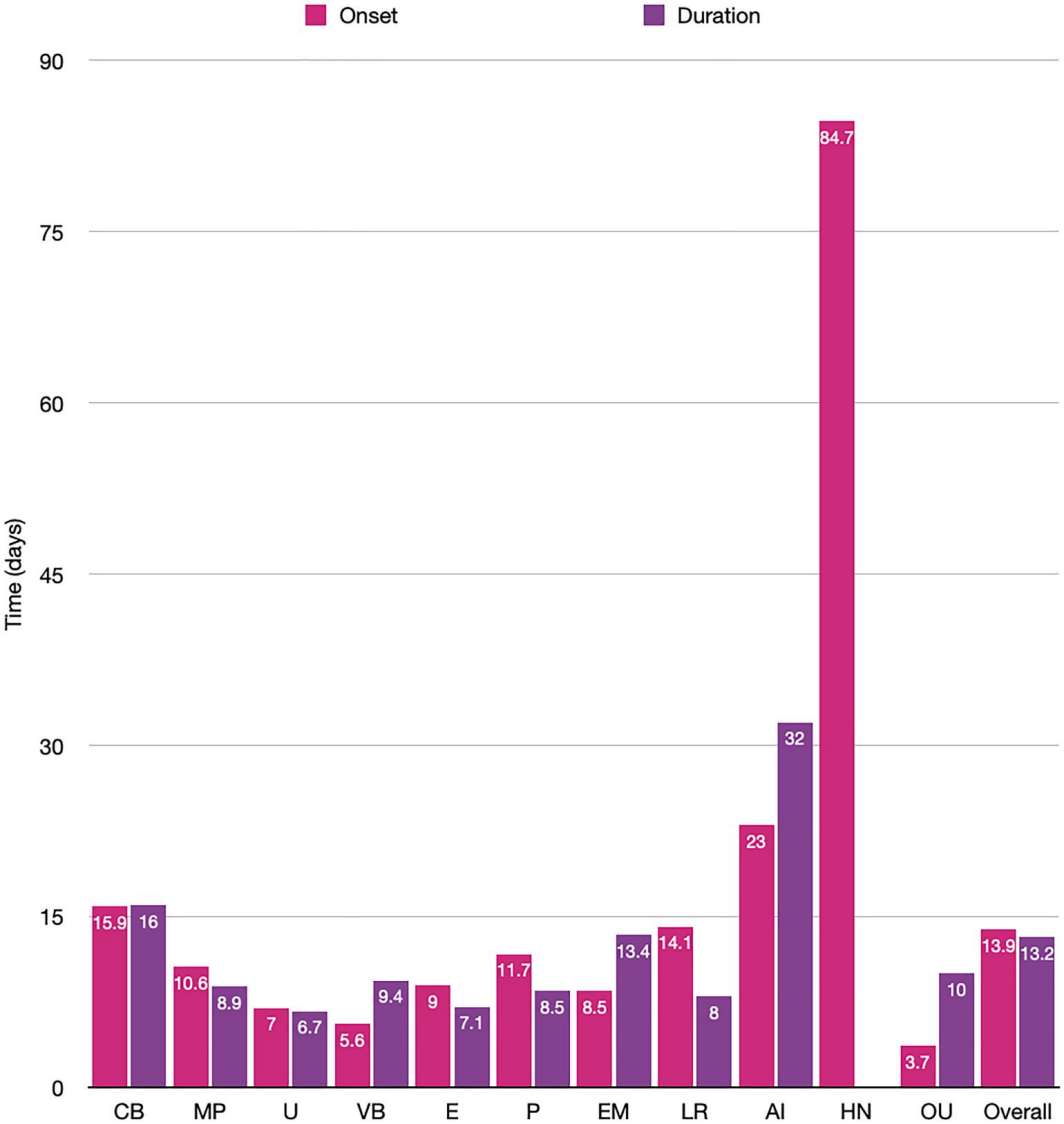
Bar chart showing rash onset with respect to systemic symptoms and duration until full resolution of skin lesions

Rash timeline varied between morphologies for both onset and duration. This is illustrated in Figure 10.

### 7.2 Sensitivity analysis

A total of 776 patients from 11 studies were included in the sensitivity analysis, of which 59.9% were outpatients, 32.3% were inpatients, 6.9% required ICU and 0.8% died. In this cohort, maculopapular was the most common rash type (27.9%), followed by chilblain‐like (23.2%). These results are illustrated in Table 4. Severity outcomes were generally quite similar to the overall group pre‐sensitivity analysis, except for the vesiculobullous cohort which was conversely associated with lower severity of COVID‐19. These results are shown in Table 5. There was not enough data for secondary outcomes to conduct a meaningful sensitivity analysis.

**TABLE 4:** Primary outcomes of rash morphology and COVID‐19 disease severity in the sensitivity analysis group

Outcome	Total	Proportion (%)	95% CI	Chi‐squared
Morphology (*n* = 776)				
(a) Chilblain‐like	180	23.2	20.3, 26.3	*p* < 0.001
(b) Maculopapular	217	27.9	24.8, 31.3	*p* < 0.001
(c) Urticaria	127	16.4	13.8, 19.2	*p* < 0.001
(d) Vesiculobullous	79	10.2	8.1, 12.5	*p* < 0.001
(e) Erythema	43	5.5	4.0, 7.4	*p* < 0.001
(f) Purpura	24	3.1	1.9, 4.6	*p* < 0.001
(g) EM‐like	1	0.1	0, 0.01	*p* < 0.001
(h) Livedo reticularis	21	2.7	1.7, 4.1	*p* < 0.001
(i) Acro‐ischaemia	15	1.9	1.1, 3.2	*p* < 0.001
(j) Hair and nail	0	0	N/A	N/A
(k) Oral ulcers	1	0.1	0, 0.01	*p* < 0.001
(L) Other	52	6.7	5.0, 8.7	
Severity (*n* = 776)				
(a) OP	465	59.9	56.4, 63.4	
(b) IP	251	32.3	29.1, 35.8	
(c) ICU	54	6.9	5.3, 8.9	
(d) RIP	6	0.8	0.3, 1.7	

**TABLE 5:** COVID‐19 severity outcomes by rash type in the sensitivity analysis group

Outcome	OR	95% CI		OR	95% CI
Chilblain‐like			Erythema		
(a) OP	17.35	8.99, 33.48	(a) OP	0.01	0.0004, 0.11
(b) IP	0.07	0.03, 0.14	(b) IP	219.27	13.44, 3578.41
(c) ICU	0.12	0.03, 0.49	(c) ICU	0.14	0.01, 2.36
(d) RIP	0.25	0.01, 4.49	(d) RIP	1.29	0.07, 23.21
Maculopapular			Purpura		
(a) OP	0.19	0.14, 0.27	(a) OP	0.26	0.11, 0.65
(b) IP	4.41	3.16, 6.15	(b) IP	0.41	0.14, 1.21
(c) ICU	1.71	0.96, 3.02	(c) ICU	20.49	8.65, 48.55
(d) RIP	5.23	0.95, 28.76	(d) RIP	2.34	0.12, 42.79
Urticaria			Livedo reticularis		
(a) OP	0.85	0.58, 1.25	(a) OP	0.32	0.13, 0.81
(b) IP	1.23	0.83, 1.83	(b) IP	2.36	0.99, 5.63
(c) ICU	1.02	0.49, 2.15	(c) ICU	4.50	1.58, 12.80
(d) RIP	0.39	0.02, 6.94	(d) RIP	19.76	3.41, 114.58
Vesiculobullous			Acro‐ischaemia		
(a) OP	3.45	1.90, 6.26	(a) OP	0.02	0.001, 0.34
(b) IP	0.34	0.18, 0.65	(b) IP	0.15	0.02, 1.12
(c) ICU	0.32	0.08, 1.35	(c) ICU	252.35	32.37, 1967.34
(d) RIP	0.67	0.03, 11.99	(d) RIP	3.74	0.20, 69.53

## 8 DISCUSSION

COVID‐19 is a viral infection characterized by respiratory symptoms, fever, myalgia, fatigue and anosmia. Aside from these classic features, many systems may also be affected, including the skin. Other coronavirus syndromes, such as severe acute respiratory syndrome (SARS) and Middle East respiratory syndrome (MERS), do not seem to result in significant skin changes. This is despite phylogenetic analysis of SARS‐CoV‐2 showing that it is closely related to both SARS‐CoV (∼79%) and MERS‐CoV (∼50%).^8^ Perhaps less is known about these viruses' disease processes as they never reached the same pandemic level as COVID‐19. However, searching the literature for cutaneous manifestations of other common respiratory viruses such as influenza yields equally few results, making COVID‐19 unique. Available data on prevalence of skin rash in COVID‐19 vary due to a number of factors, including those related to both the patient and the assessor. For example, factors such as whether the patient was in the outpatient setting or admitted to hospital, in addition to whether the rash was self‐reported, documented by a trained Dermatologist, or by another type of physician all contributed to variable levels of estimated prevalence. A previous review summarized the pooled prevalence from studies with non‐selective reporting of skin rash in COVID‐19 infection and estimated it to be 5.69%.^5^ Extrapolating this and applying it to the 250 million people that have been infected so far, it is likely that more than 14 million people have, or will soon be affected by cutaneous manifestations of COVID‐19.^1^ This review examined a proportion of that population, with efforts made to account for confounders such as age, medications, pre‐existing dermatological or rheumatological disease, iatrogenic rashes and other secondary pathogens.

Two thousand fifty‐six patients with cutaneous manifestations secondary to COVID‐19 were identified from 240 studies. Most studies were from Europe or America, but others from Asia, South America, Australia and Africa were also included. The population had an equal proportion of each gender, but seemed to have an unequal distribution of ethnicities. Patients referred to as ‘White’ were overrepresented and accounted for 83% (*n* = 293/353) of those with information regarding race. This is likely to be reflective of the general demographics of the countries publishing the most articles. Notably, the vast majority of studies mentioning race were published from America, where 76% of people are classified as ‘White’ as per the US census.^9^ Age ranged from 0 to 100, with a mean of 35 years. This is younger than in previously published systematic reviews, which is probably reflective of the higher proportion of chilblain‐like lesions included in this study as they seemed to occur more frequently in younger patients, with a mean of 21.5 years (SD ± 10.8). With chilblain‐like lesions excluded, the mean age of patients in this study increases to 48.5 (SD ± 16.3), which is very similar to previously published reviews.^6^

**FIGURE 11:**
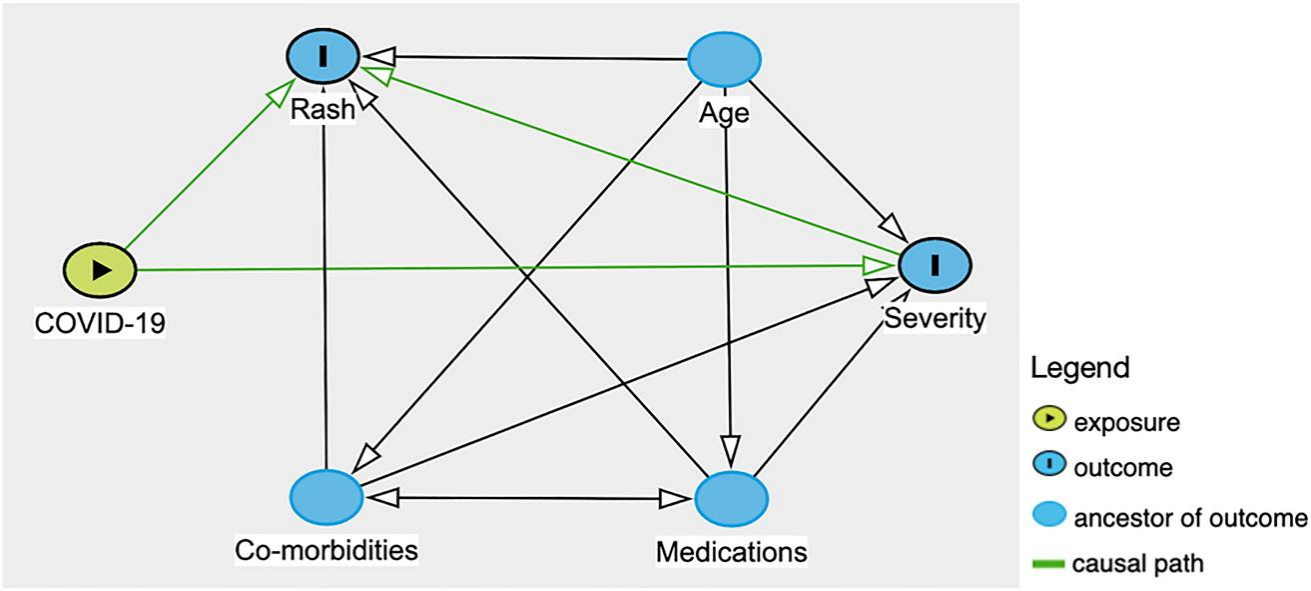
Direct acyclic graph illustrating causal inference and potential confounders to rash and COVID‐19 severity

**FIGURE 12:**
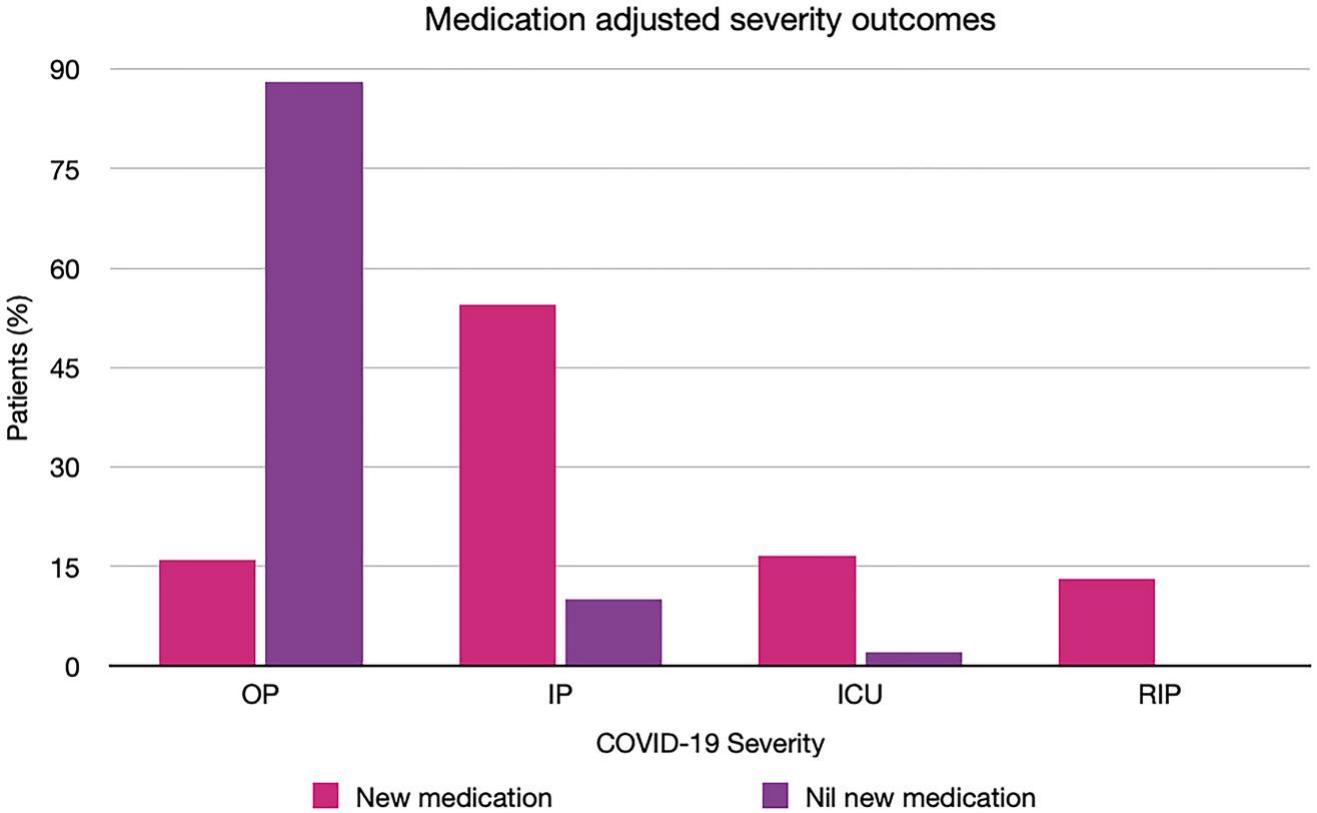
Bar chart demonstrating proportions of patients within each severity outcome for those with new medications or no new medications

Chilblain‐like lesions were by far the most common rash type seen in the main part of this review (54.2%), which is in contrast to studies published earlier in the pandemic.^6^, ^7^ Increased awareness of their association with COVID‐19, augmented by the media coverage it has received, is likely the cause for higher levels of reporting, rather than increasing incidence. A possible overrepresentation of these lesions in the literature might explain why we saw a lower proportion of them in the sensitivity analysis group. When compared to other rashes, it was the type that was most significantly associated with outpatient level severity (OR 35.36 [95% CI 23.58, 53.03]). All other rash morphologies were negatively associated with outpatient level of COVID‐19 care, except for erythema multiforme‐like, which did not have a statistically significant association with any severity outcome, and vesiculobullous, which was associated with mortality (OR 6.07 [95% CI 1.75, 21.07]) in the overall group, but outpatient severity (OR 3.45 [95% CI 1.90, 6.26]) in the sensitivity analysis group and otherwise no significant relationship with the other levels of severity. However, the data on this rash type came from only two papers, with one of them being made up entirely of outpatients, which may have contributed to inconsistent results. Overall, the results indicate that among patients with a COVID‐19 related rash, those with chilblain‐like lesions are likely to not require care beyond the outpatient setting, while those with acro‐ischaemia, purpura, livedo reticularis, urticaria, maculopapular rash, erythema, hair and nail changes and oral ulcers are all likely to require admission to hospital at some point in their COVID‐19 disease course. There was a clear relationship between some rash morphologies and a higher degree of severity of COVID‐19. Having acro‐ischaemia (OR 34.01 [95% CI 16.62, 69.57]), purpura (OR 7.22 [95% CI 4.03, 12.92]), livedo reticularis (OR 5.57 [95% CI 3.02, 10.30]), maculopapular rash (OR 2.71 [95% CI 1.74, 4.21]) or erythema (OR 2.50 [95% CI 1.25, 5.00]) meant patients had a higher risk of being transferred to ICU versus other rash types. Among those, acro‐ischaemia (OR 25.66 [95% CI 10.83, 60.79]) and livedo reticularis (OR 10.71 [95% CI 4.76, 24.13]) were additionally associated with a greater rate of mortality from COVID‐19. A number of potential confounders exist, including age, medications and comorbidities, as demonstrated in the DAG (Figure 11). To minimize the effect of comorbidities on interpretation of the results, patients with a history of Dermatological and Rheumatological conditions were excluded. Medications may have an effect on both rash and COVID‐19 severity. Full medication data was available for only 578 patients. The medications were commenced to improve patient morbidity and mortality, but the fact that they were introduced in the first place is also an indication of COVID‐19 severity. Hydroxychloroquine was the most common new medication reported, but its use in COVID‐19 is controversial. It has been in existence for many years and is commonly prescribed for conditions such as Systemic Lupus Erythematosus and for malaria prophylaxis. As a result, it is a well‐understood drug and has shown several mechanisms, including the inhibition of inflammatory cytokines such as interleukin‐1 (IL‐1), IL‐6 and Tumour Necrosis Factor‐alpha (TNFa).^10^ On this basis, it was thought that it might have beneficial effects in the treatment of COVID‐19 and early in vitro studies supported this theory.^11^, ^12^ However, subsequent randomized controlled trials have confirmed that hydroxychloroquine does not improve clinical status and is also associated with an increased rate of adverse events.^13^, ^14^, ^15^, ^16^ Among the adverse events that it is known to cause are retinopathy, QT prolongation and perhaps most significantly in this context; rash.^17^ A review of 3578 patients who experienced adverse events due to hydroxychloroquine found that 689 (19.3%) had dermatological effects.^18^ This included 21 distinct dermatologic reactions, the most common being drug rash (358 cases).^18^ Among those described as having a drug rash, maculopapular, erythematous and urticarial were stated as being the most common.^18^ In our review, those three rash morphologies had high rates of new medications (93.9%, 70.8% and 78.7%, respectively), along with the two rash types with the strongest association for worse severity outcomes, acro‐ischaemia (94.4%) and livedo reticularis (93.5%). This may be coincidental, reflective of worse severity of COVID‐19, or indicative of possible drug reactions. After hydroxychloroquine, the next most common new medications were azithromycin and antiviral drugs such as lopinavir/ritonavir, all of which are also known to cause a wide range of cutaneous drug reactions.^19^, ^20^ Clearly, medications are a significant confounder when examining the relationship between rash type and disease severity. However, fully accounting for it is difficult, as excluding those with recent new drugs would disproportionately affect the four severity outcomes. In this review, there were 299 patients with COVID‐19 severity data who were also known to have no recent medication changes. Two hundred sixty‐two (88%) of them had outpatient level severity, while 29 (10%) were inpatients, 7 (2%) were admitted to ICU and only 1 (0.3%) died. There were 145 patients with severity data who had been commenced on a new drug before rash onset. This included 23 (15.9%) outpatients, 79 (54.5%) inpatients, 24 (16.6%) patients in ICU and 19 (13.1%) patients who passed away. The overrepresentation of lower severity outcomes in the cohort with no new medications is illustrated in Figure 12. Ideally, a prospective study with a treatment and a control arm would examine this relationship further, but it would pose logistical and ethical challenges.

**FIGURE 13:**
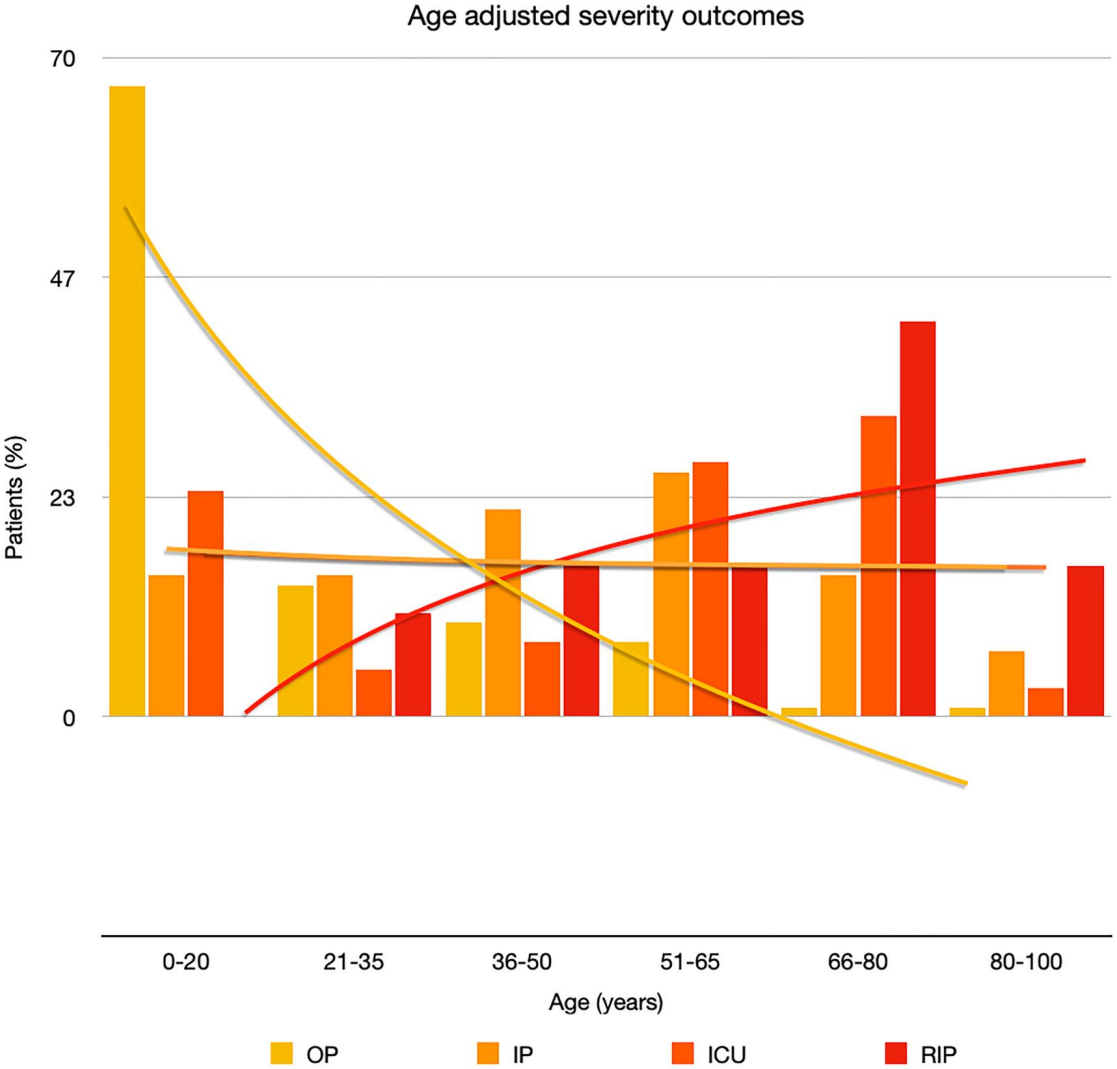
Bar chart showing a linear scale of percentage of patients as a proportion of total numbers within each severity outcome, and organized by age group for all patients in the study. Logarithmic trend lines included correspond to matching colour

**FIGURE 14:**
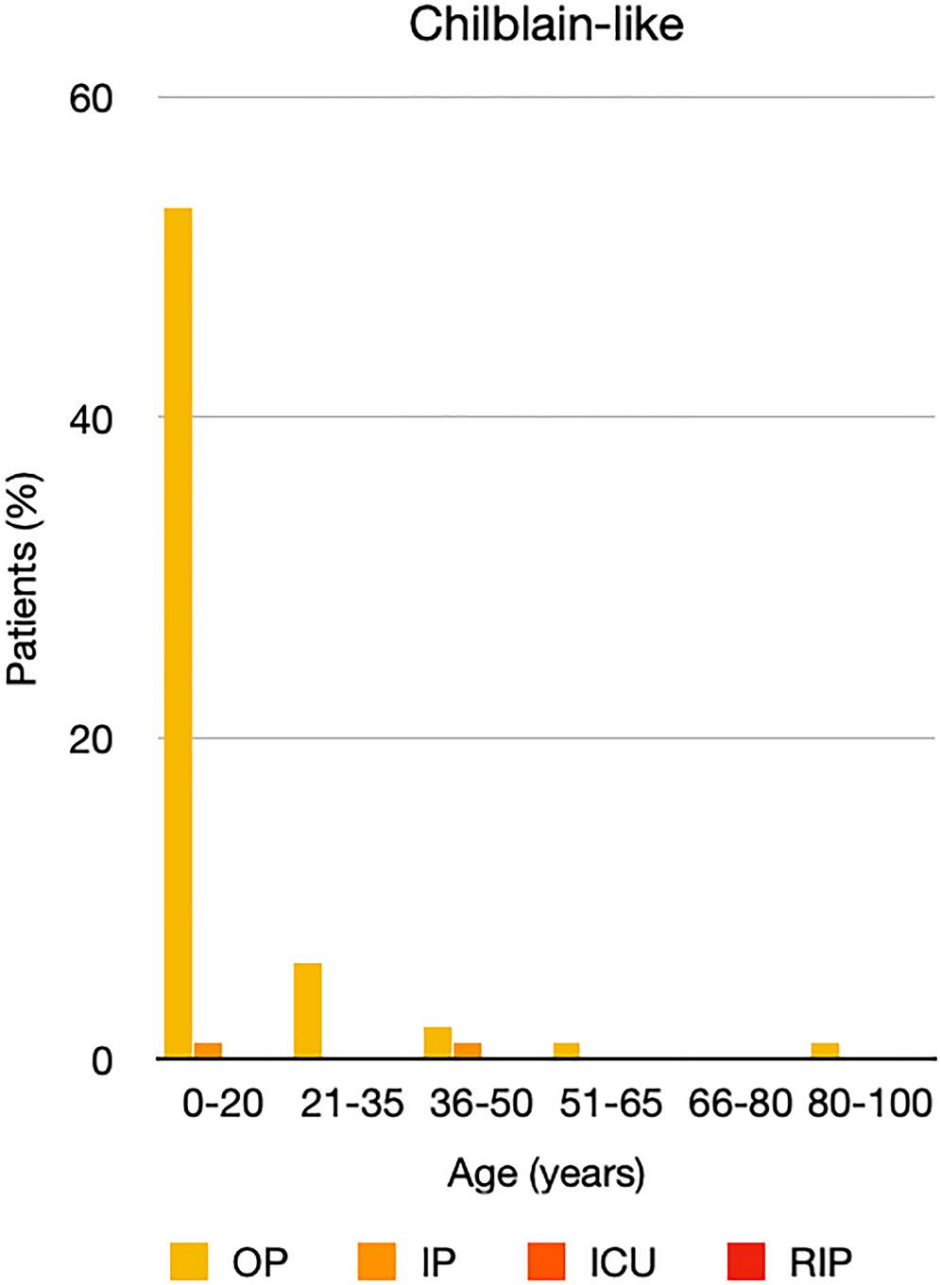
Bar chart showing % of patients as a proportion of total numbers within each severity outcome, by age group for those with chilblain‐like lesions

**FIGURE 15:**
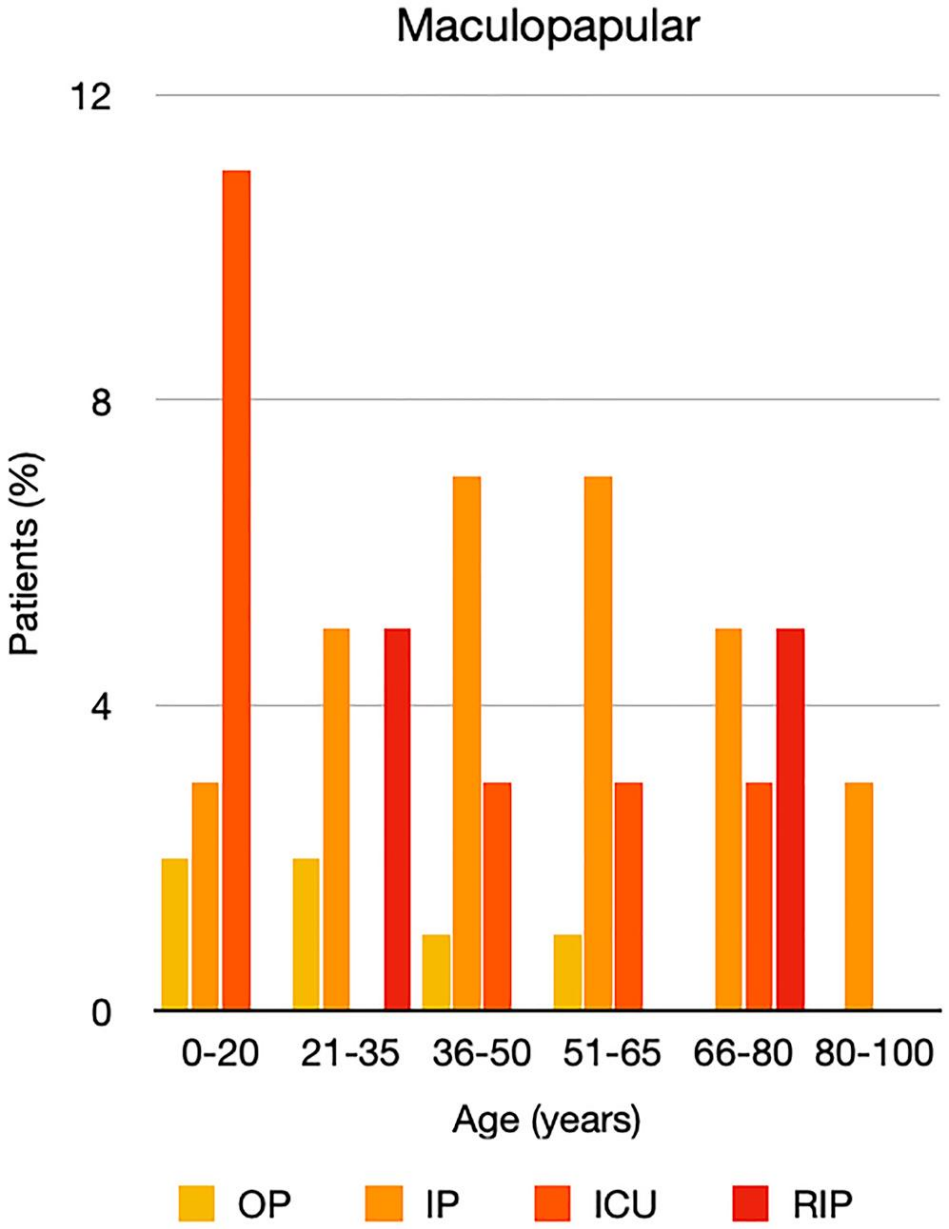
Bar chart showing % of patients as a proportion of total numbers within each severity outcome, by age group for those with maculopapular rash

**FIGURE 16:**
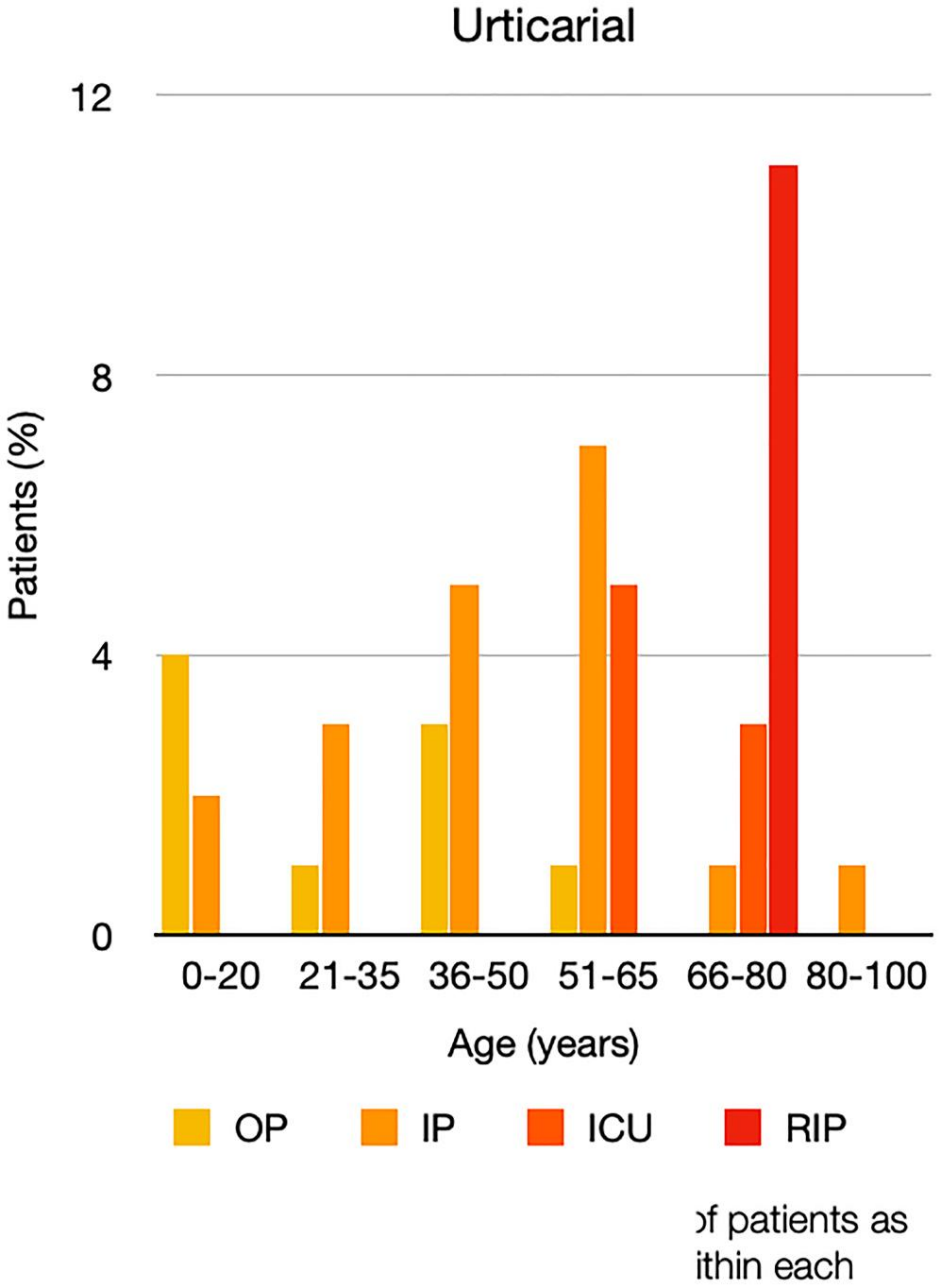
Bar chart showing % of patients as a proportion of total numbers within each severity outcome, by age group for those with urticarial rash

**FIGURE 17:**
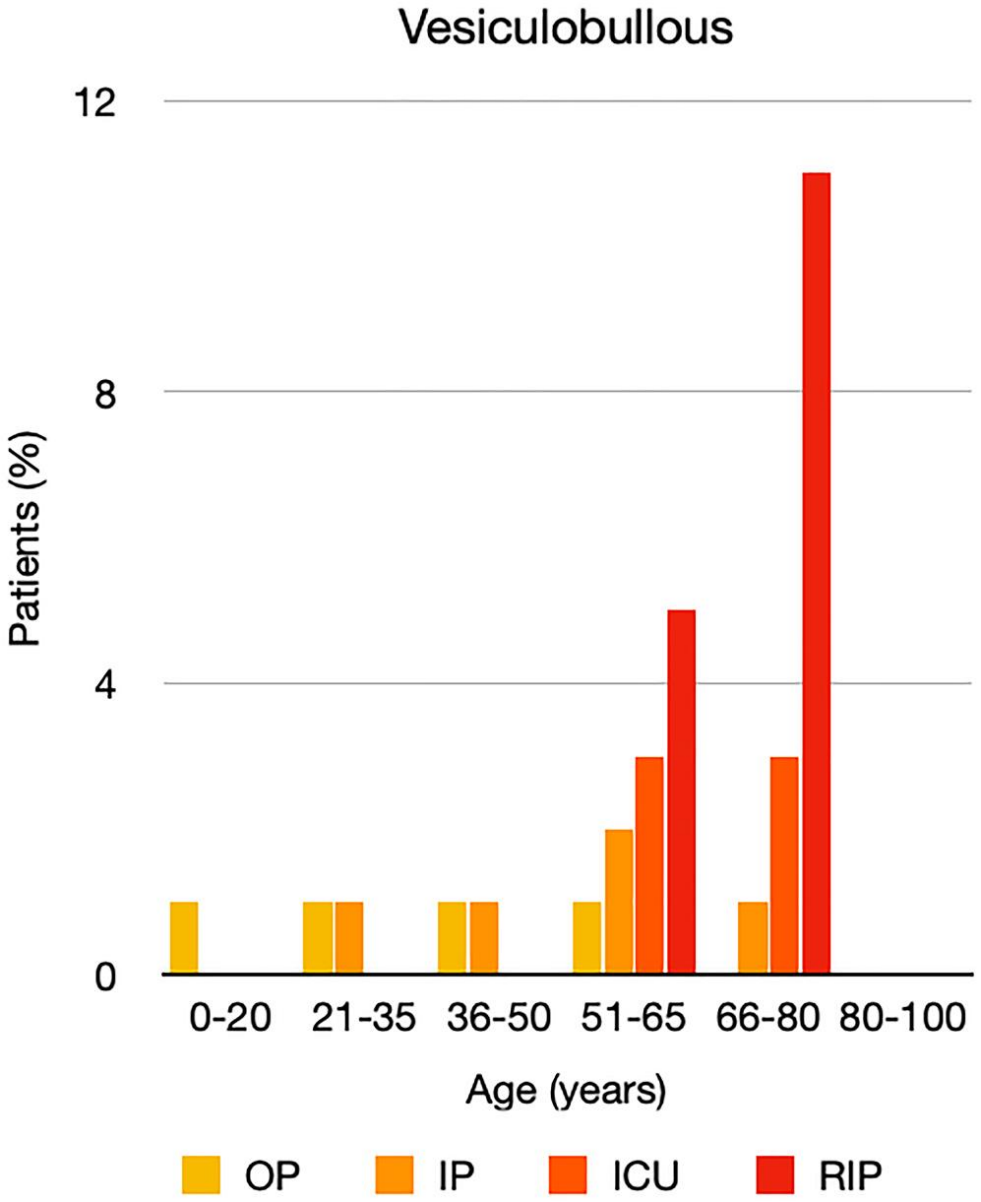
Bar chart showing % of patients as a proportion of total numbers within each severity outcome, by age group for those with vesiculobullous rash

**FIGURE 18:**
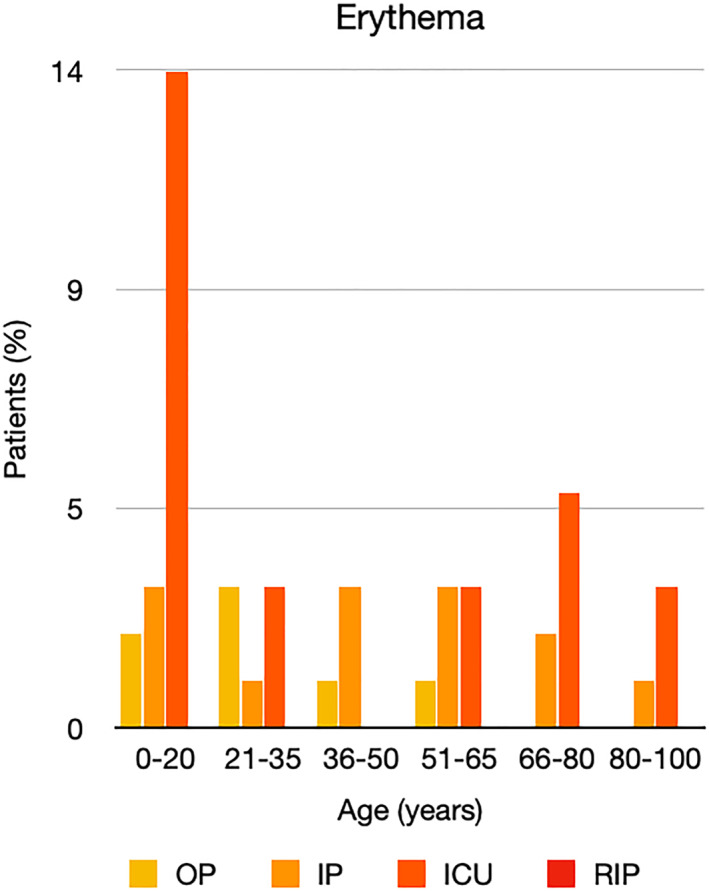
Bar chart showing % of patients as a proportion of total numbers within each severity outcome, by age group for those with erythematous rash

**FIGURE 19:**
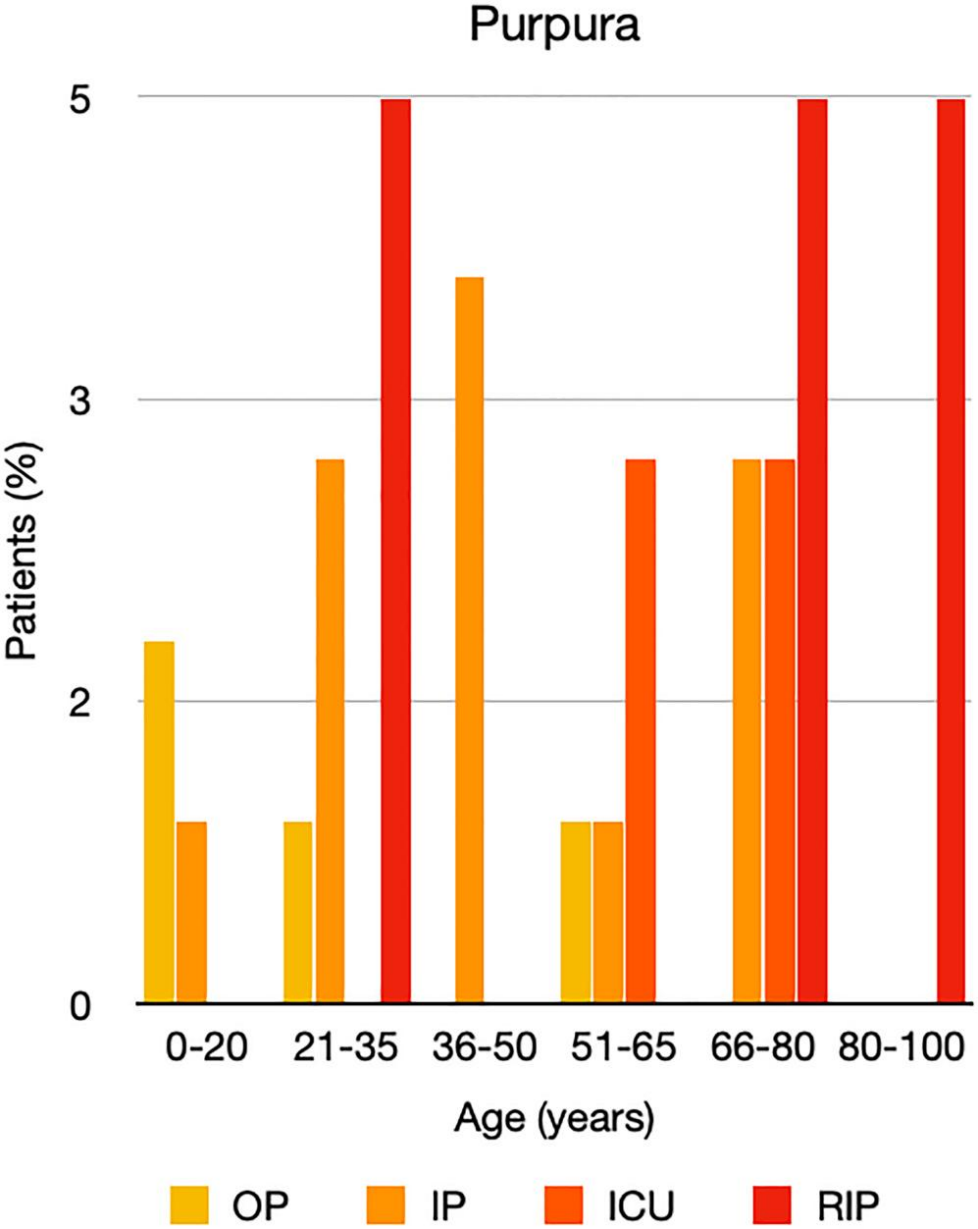
Bar chart showing % of patients as a proportion of total numbers within each severity outcome, by age group for those with purpuric rash

**FIGURE 20:**
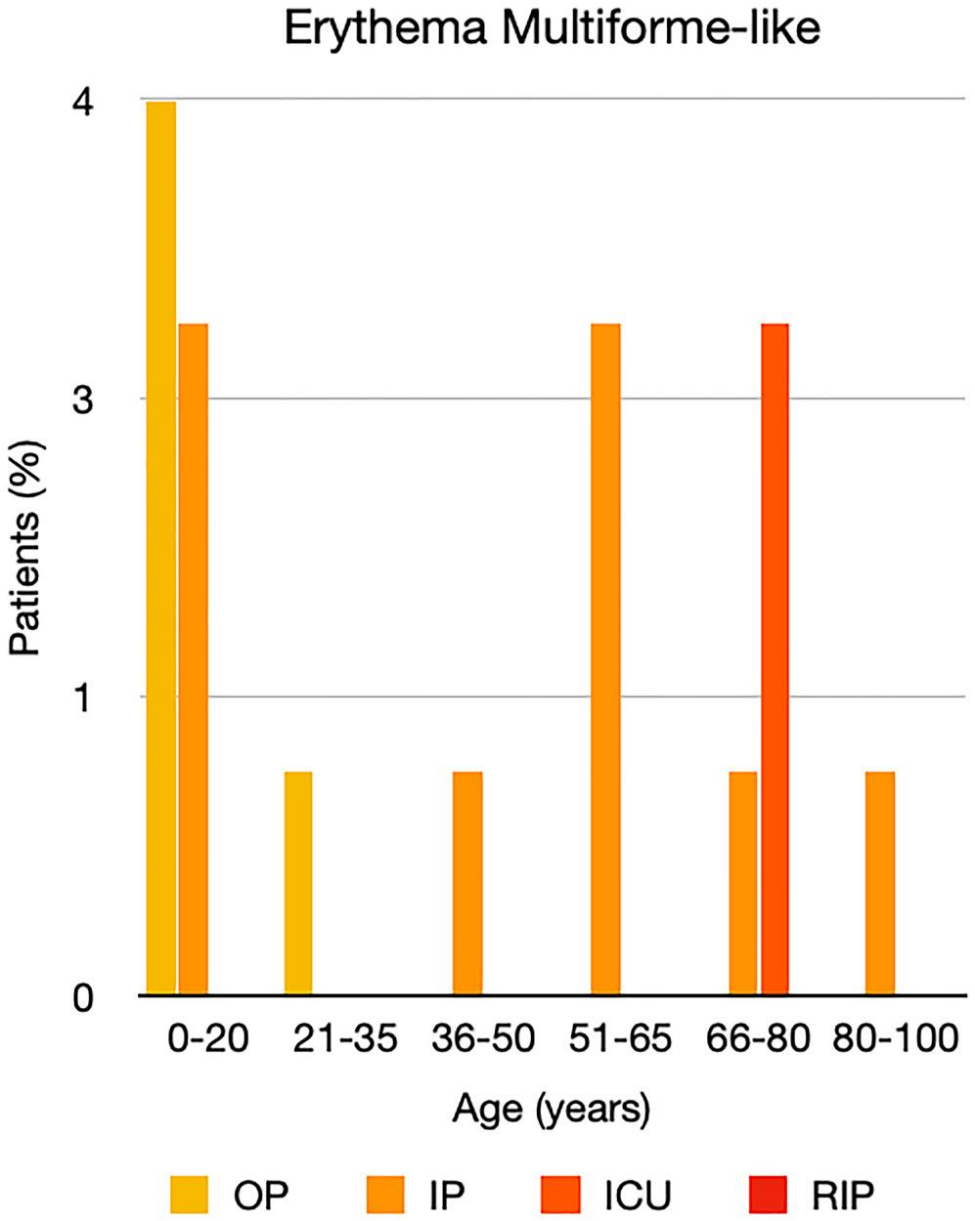
Bar chart showing % of patients as a proportion of total numbers within each severity outcome, by age group for those with erythema multiforme‐like rash

**FIGURE 21:**
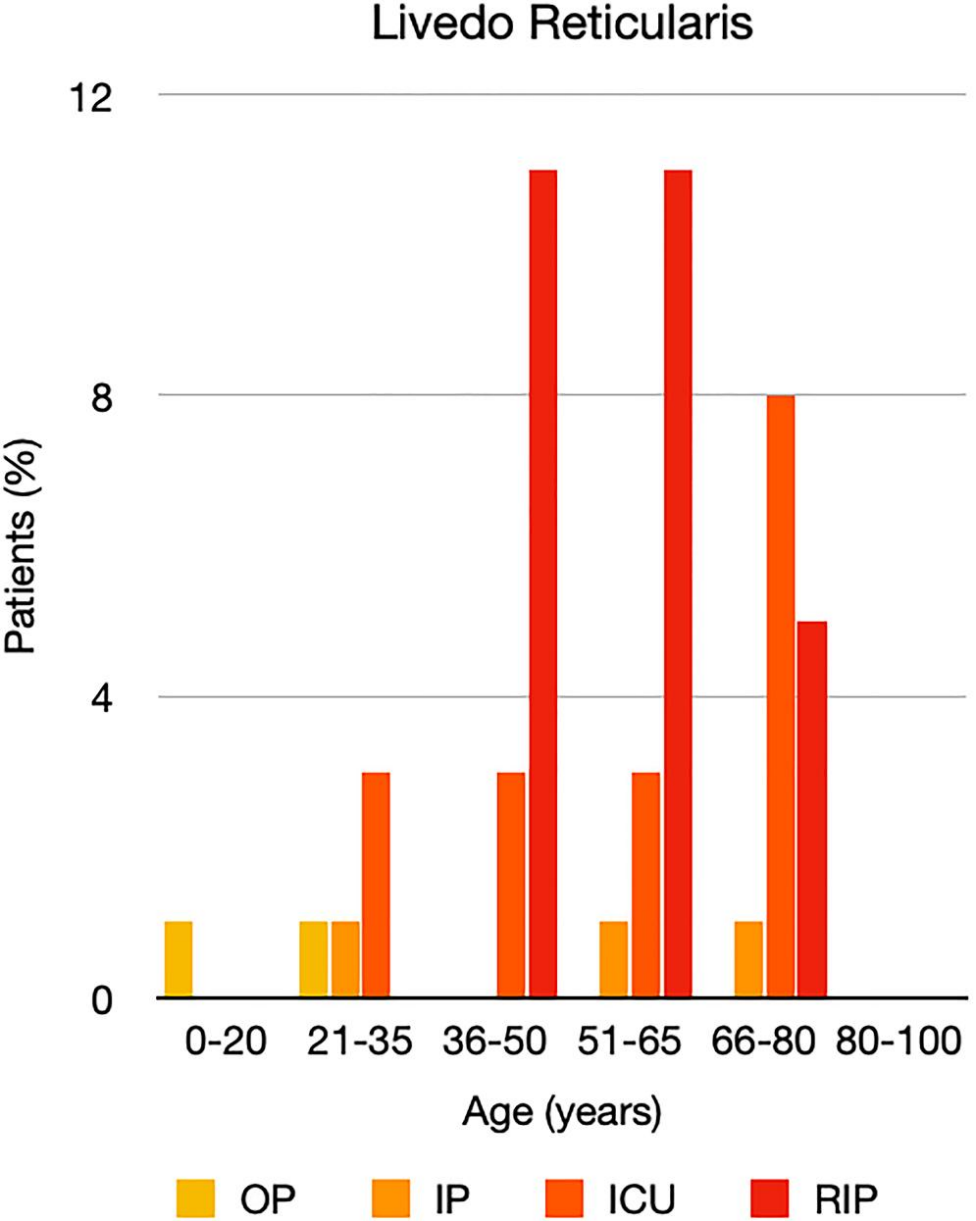
Bar chart showing % of patients as a proportion of total numbers within each severity outcome, by age group for those with livedo reticularis

**FIGURE 22:**
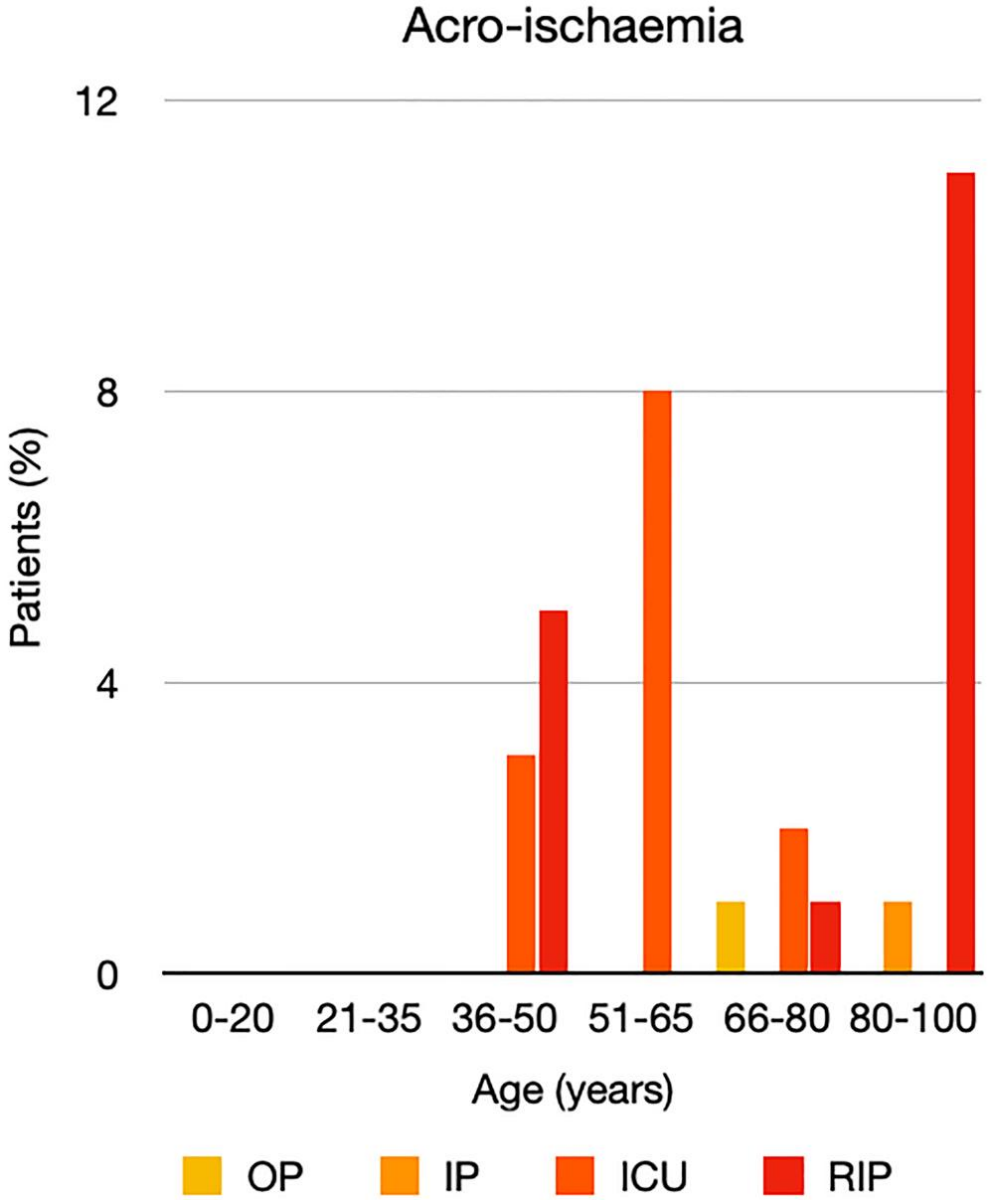
Bar chart showing % of patients as a proportion of total numbers within each severity outcome, by age group for those with acro‐ischaemia

**FIGURE 23:**
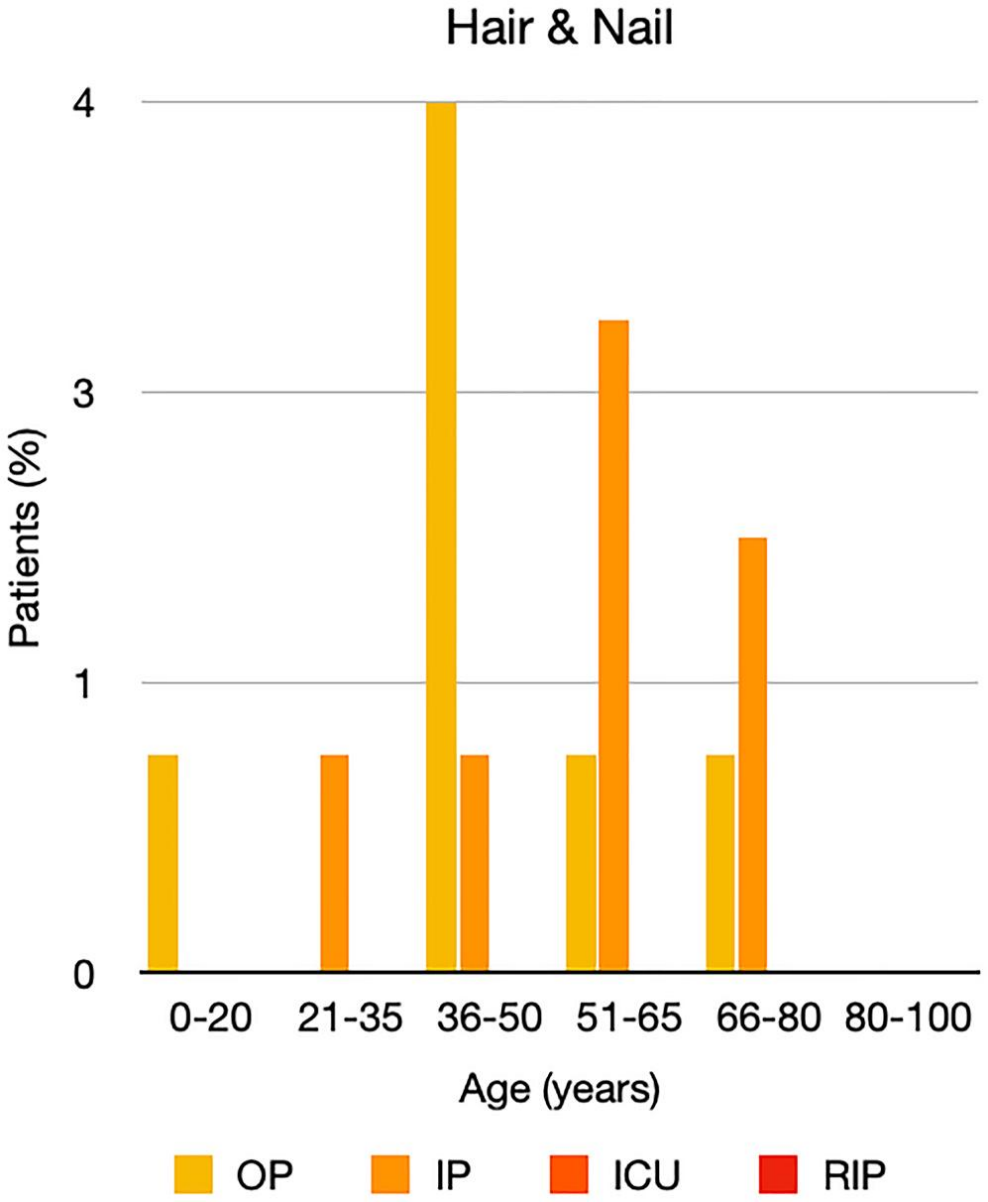
Bar chart showing % of patients as a proportion of total numbers within each severity outcome, by age group for those with hair and nail changes

**FIGURE 24:**
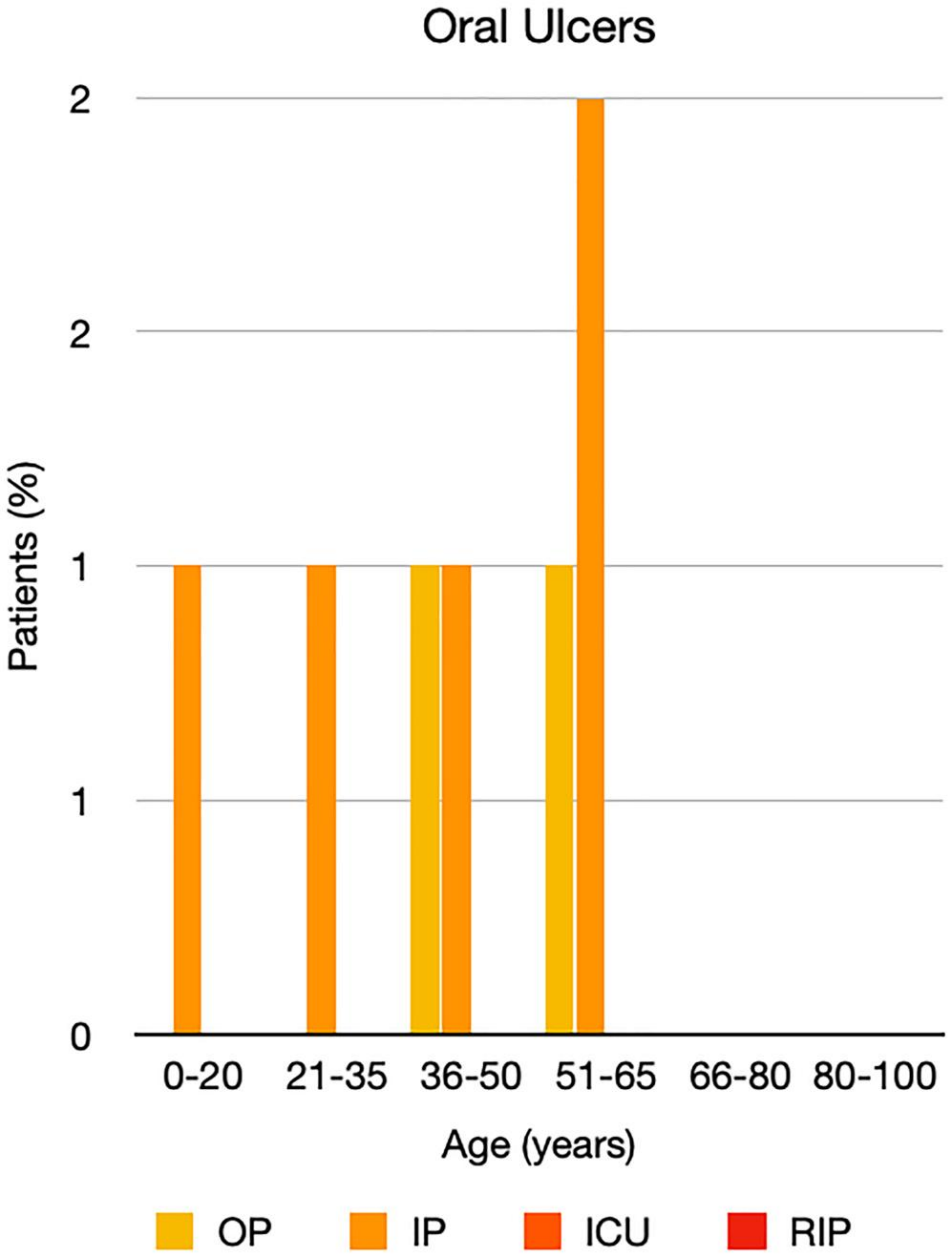
Bar chart showing % of patients as a proportion of total numbers within each severity outcome, by age group for those with oral ulcers

**FIGURE 25:**
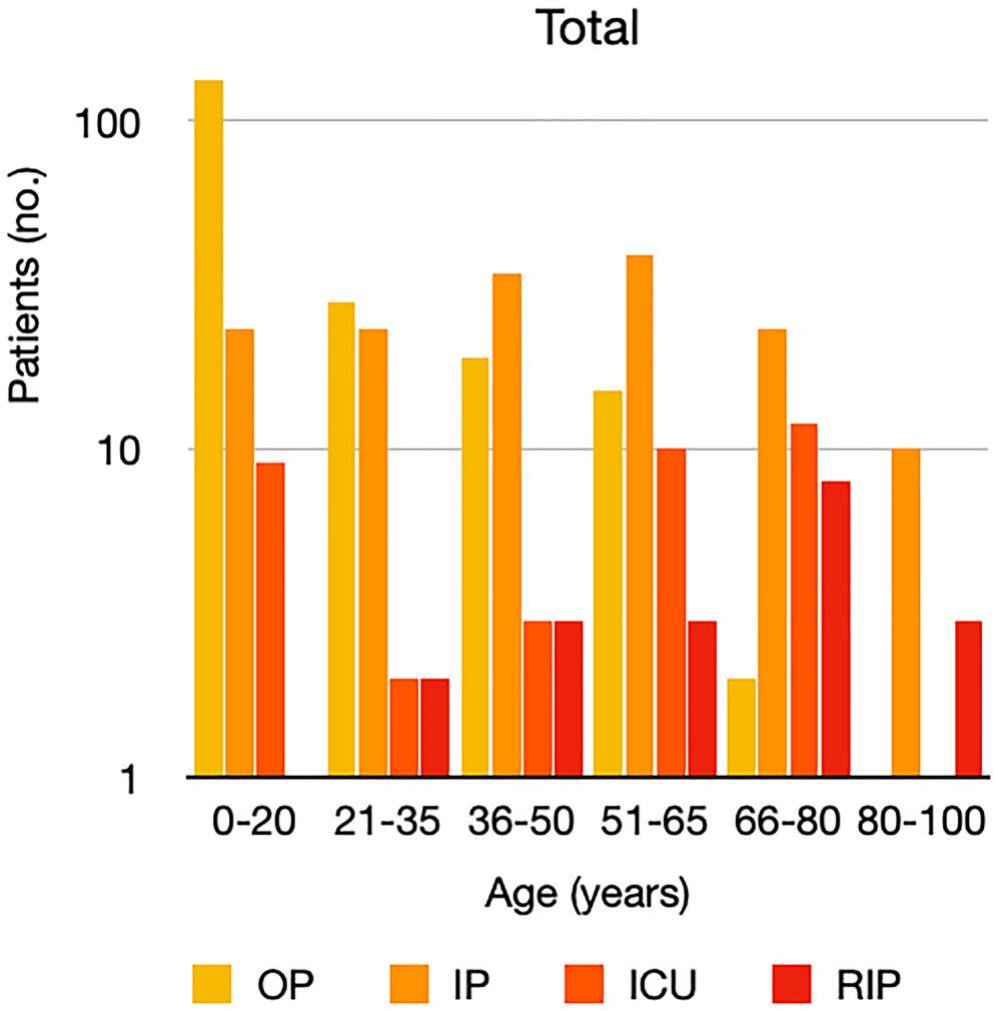
Bar chart on a logarithmic scale showing total number of patients within each severity outcome, by age group for all patients

It is well known that worse COVID‐19 outcomes are seen in those with older ages, independent of other factors such as comorbidities.^21^ When corrected for age, the patients in this review seemed to follow that trend, with the proportion of those with worse severity comparatively increasing with age. This is depicted in Figure 13. As age increases, so does the mortality rate while conversely, the outpatient rate significantly decreases. The trend lines for inpatient and ICU severity levels remain relatively stable throughout all age ranges. Examining the ICU data further shows that the proportion of patients in the lowest age group (0–20 years) is relatively high, while the proportion in the oldest age group (80–100 years) is comparatively low. This may be explained by those being admitted to ICU at a young age having a higher chance of recovering and being discharged home than those of older ages, who have a greater mortality risk. This is supported by the mortality figures in those age groups. Otherwise, the proportion in the ICU group increased with advancing age. In the inpatient group, proportions increased up until the 51–65 years age group, which is probably reflective of more patients requiring a higher level of care or dying in the older cohorts. Age‐adjusted severity outcome graphs for each rash morphology demonstrate this (Figures 14, 15, 16, 17, 18, 19, 20, 21, 22, 23, 24, 25). Interestingly, the two rash morphologies that were most strongly associated with poor outcomes, acro‐ischaemia and livedo reticularis, were the two groups with the highest mean ages, 58.43 and 55.60 years, respectively. Conversely, the cutaneous manifestation associated with the lowest severity outcome, chilblain‐like lesions, had by far the lowest mean age; 21.46 years. If age is a known powerful determinant of outcome and rash is a significant indicator of severity, as shown in this review, then there could be a relationship between the two factors. It seems that in certain age groups, patients are either at an increased or decreased risk of severe disease. Mirroring this, certain rash morphologies are more frequently seen in some age cohorts. Cutaneous signs could therefore be used as significant prognostic indicators and inform the treating clinician on the likelihood of the patient deteriorating. This could be very useful for planning and allocation of resources in addition to potentially improving patient outcomes if deteriorations are acted upon earlier or even prevented altogether.

Of those with full diagnostic method data available in this review, approximately a third of the patients had either a positive swab or serum antibodies, while the remainder either tested negative or were presumed to be infected. A multitude of different factors exists that affect the likelihood of a person being referred for a COVID‐19 test, aside from respiratory symptoms. In the early stages of epidemics caused by a novel infectious pathogen, testing is typically biased towards the more severe cases due to limited diagnostic capabilities.^22^ Limited resources usually result in more stringent inclusion criteria for testing, for example, a specific combination of clinical symptoms in addition to close contacts and travel history. These restrictive criteria may result in omission of infections with a low disease severity, such as those with chilblain‐like lesions. This group was frequently paucisymptomatic, with chilblain‐like lesions being the only symptom in 464 patients in this review and as a result, it was also the group with by far the highest number of patients not tested (*n* = 505). There are also a significant proportion of patients who become infected, but remain asymptomatic for the entire disease course. A large review of high‐quality data estimated this figure to be at least one third of all infected patients. In our study, rash was the only symptom in 20.9%.^23^ Rash may also go unnoticed and underreported in the absence of dermatology involvement, which is demonstrated by the fact that prevalence studies involving dermatology have reported higher rates of cutaneous findings than those where patients were not examined by a skin specialist.^2^, ^3^, ^4^, ^5^ Unfortunately, not all patients included in this study were examined by a dermatologist. Aside from testing availability, significant challenges exist regarding test processing and interpretation. The urgent need to implement and rapidly expand testing led to the development of multiple different assays. While these assays have been shown to produce generally comparable results, slight differences applied on a grand scale can have significant outcome effects.^24^ The testing methods available are also far from perfect, with one systematic review reporting false negative rates up to 29%.^25^ Further studies with standardized diagnostic methods are required.

Complete data on dermatological symptoms was available for 700 patients. Pruritus was the most commonly reported symptom (35.1%), followed by pain (16.4%), then burning (4.7%). 34.1% of patients had asymptomatic skin lesions. These symptoms can carry significant physical morbidity in addition to aesthetic implications. Our knowledge of the morbidities associated with COVID‐19 is improving. For example, long COVID has been shown to cause significant and prolonged disability, and we are likely to see long‐term respiratory implications in those who suffered severe pulmonary damage.^26^ Additionally, reports are increasing of children developing a multisystem inflammatory syndrome, similar to Kawasaki's syndrome, with potential for long‐term cardiac damage.^27^ Notably, 17 patients in this review fulfilled the criteria for multisystem inflammatory syndrome in children (MIS‐C). Given that COVID‐19 has the potential to affect multiple different systems, it can lead to a wide range of complications. However, the effect of dermatological symptoms on morbidity can be significant and should not be underestimated.

Timeline of cutaneous manifestations with respect to systemic symptoms was also examined. Oral ulcers had the earliest onset, with a mean of 3.7 days, while hair and nail changes were the latest. They were not seen until approximately 3 months after the COVID‐19 episode, which is in keeping with hair and nail life cycles. Otherwise, most rash types appeared within the ‘inflammatory phase’ window between 7 and 12 days. The morphologies associated with more severe COVID‐19 disease, namely purpura (11.7 days), livedo reticularis (14.1 days) and acro‐ischaemia (23 days), all seemed to appear later in the disease course, as did chilblain‐like lesions (15.9 days). Rash duration varied, but lasted for between 7 and 14 days for most subtypes. Urticaria resolved the quickest (6.7 days) and acro‐ischaemia lasted the longest (32 days). There was no data for hair and nail change duration. Data on duration should be interpreted with caution as it is confounded by both COVID‐19 treatment and rash specific therapies, which were not examined in detail in this review. Cutaneous manifestations were also occasionally seen as the first or only symptom. Notably, chilblain‐like lesions were the only symptom in 464 patients, far more than any other rash type, which is in keeping with its known paucisymptomatic nature and associated high rate of negative testing. The appearance of an otherwise unexplained skin change may have potential use in aiding the diagnosis of COVID‐19, but given that this study only included patients with COVID‐19 and a rash, it is unable to comment further on diagnostic value.

The sensitivity analysis showed similar results in the primary outcomes to the initial study, indicating that the sample is a reasonably accurate representation of the true population. There was insufficient data on the secondary outcomes in the sensitivity analysis group to allow a fair comparison.

## 9 LIMITATIONS

There are several limitations to this study. To date, there is no standardized criteria for classifying morphological rash types in COVID‐19. As a result, some patients were counted in more than one category, which may skew results. There is also a need for standardized criteria for diagnosing COVID‐19, as there was variability between methods in this review and a number of patients never received formal testing. Case reports accounted for a significant proportion of the studies included. There was likely to be some reporting bias, specifically with regard to chilblain‐like lesions due to its controversial relationship with COVID‐19. There was significant heterogeneity among studies and as a result, complete data on secondary outcomes were not always available. Patients with complete data were sampled to estimate proportions; however, this sample may not be accurately representative of the whole population. Results in these outcomes should be interpreted with caution. Confounders to primary outcome results include age, medications and comorbidities, which were not examined in this review. As studies with non‐selective reporting of cutaneous manifestations were included, this review cannot comment on the use of rash in the diagnosis of COVID‐19.

## 10 CONCLUSION

In conclusion, there are a wide range of cutaneous manifestations associated with COVID‐19. Maculopapular rash and chilblain‐like lesions were the most common morphologies, with the latter found to be strongly associated with a paucisymptomatic disease course and a low severity of COVID‐19. Conversely, skin changes such as acro‐ischaemia, livedo reticularis and purpura may be useful indicators of a higher severity of COVID‐19 disease. Rash as a clinical feature might therefore be helpful in determining COVID‐19 prognosis among those with skin changes, but this is confounded by age, medications and comorbidities. These cutaneous manifestations can also cause symptoms such as pain, burning and pruritus, which carry a morbidity burden and should not be overlooked. While this study has several limitations, including significant heterogeneity, the data presented is still useful in progressing the understanding of COVID‐19, specifically in the context of dermatology. The skin has the potential to play an important role in multiple aspects of the diagnosis and treatment of COVID‐19.

## CONFLICT OF INTEREST

The authors declare that there are no conflict of interests.

## AUTHOR CONTRIBUTIONS

Zack Holmes: Conceptualization; data curation; formal analysis; investigation; methodology; project administration; writing ‐ original draft; writing – review & editing. Ashling Courtney: Data curation; methodology; writing – review & editing. Marc Lincoln: Formal analysis; methodology; writing – review & editing. Richard Weller: Supervision; writing – review & editing.

## Data Availability

The data that support the findings of this study are available from the corresponding author upon reasonable request.
